# Supplementary material for: Twofold rigidity activates ultralong organic high-temperature phosphorescence
Source: Nat Commun. 2024 Feb 10;15:1269. doi: 10.1038/s41467-024-45678-1 (PMC10858902; doi:10.1038/s41467-024-45678-1)
Supplement: Supplementary file 1 — Supplementary Information [file 41467_2024_45678_MOESM1_ESM.pdf]

Supplementary Information

Twofold Rigidity Activates Ultralong Organic High-Temperature Phosphorescence

Kaijun Chen[‡a], Yongfeng Zhang[‡b], Yunxiang Lei[*a], Wenbo Dai[a], Miaochang Liu[a], Zhengxu Cai[b], Huayue Wu[a], Xiaobo Huang[*a] and Xiang Ma[*c]

[a] School of Chemistry and Materials Engineering, Wenzhou University, Wenzhou 325035, P. R. China

[b] School of Materials Science & Engineering, Beijing Institute of Technology, Beijing 10081, P. R. China

[c] Key Laboratory for Advanced Materials and Feringa Nobel Prize Scientist Joint Research Center, Frontiers Science Center for Materiobiology and Dynamic Chemistry, School of Chemistry and Molecular Engineering, East China University of Science and Technology, Meilong Road 130, Shanghai 200237, P. R. China

E-mail: yunxianglei@wzu.edu.cn (Y. Lei); xiaobhuang@wzu.edu.cn (X. Huang); maxiang@ecust.edu.cn (X. Ma)

[‡] These authors contributed equally.

Supplementary Methods

^{1}H and ^{13}C NMR spectra were carried out by a Bruker ARX500 spectrometer with $CDCl_3$ as the solvent. UV-vis absorption spectra were measured by a Persee TU-1901 spectroscopy. Fluorescence spectra were measured by a Hitachi F-7000 spectrophotometer. Phosphorescence spectra were measured by a FLS920 lifetime and steady state spectrometer. X-Ray crystal structure analyses were conducted on a Bruker-AXS SMART APEX2 CCD diffractometer. Solid-state emission quantum yields were collected on a FluoroMax-4 (Horiba Jobin Yvon) fluorimeter equipped with integrated sphere. The T_g values of host films were measured by differential scanning calorimetry (DSC) using Mettler DSC 3+ with a heating rate of 10 K min^{-1} under nitrogen. The polymer host molecules (PVP, PVA, and PA66) and the guest molecules cBCZ and cgBCZ were commercially available.

9*H*-Dibenzo[*a*,*c*]carbazole (BCZ). White solid (331 mg, 62% yield). ^{1}H NMR (500 MHz, $CDCl_3$) δ: 8.80 (d, *J* = 8.0 Hz, 1H), 8.76 (d, *J* = 7.5 Hz, 3H), 8.54 (d, *J* = 7.5 Hz, 1H), 8.01 (d, *J* = 7.5 Hz, 1H), 7.77 (t, *J* = 7.5 Hz, 1H), 7.67-7.59 (m, 4H), 7.47-7.40 (m, 2H) ppm. ^{13}C NMR (126 MHz, $CDCl_3$) δ: 138.2, 133.8, 130.0, 127.4, 127.0, 126.7,

126.2, 124.8, 124.0, 123.82, 123.75, 123.65, 122.3, 121.9, 120.8, 120.7, 111.4 ppm. HRMS (ESI) m/z: $[M-H]^-$ calculated for $C_{20}H_{12}N$, 266.09752; found, 266.09818.

9-Methyl-9*H*-dibenzo[*a,c*]carbazole (BCZ-Me). White solid (247 mg, 88% yield). ^1H NMR (500 MHz, $CDCl_3$) δ: 8.87-8.82 (m, 2H), 8.75 (d, *J* = 8.5 Hz, 1H), 8.67-8.60 (m, 2H), 7.77-7.74 (m, 1H), 7.67-7.63 (m, 2H), 7.60-7.56 (m, 2H), 7.50 (t, *J* = 7.0 Hz, 1H), 7.41 (t, *J* = 7.0 Hz, 1H), 4.32-4.31 (m, 3H) ppm.^{13}C NMR (126 MHz, $CDCl_3$) δ: 139.7, 133.6, 129.8, 128.9, 126.2, 125.8, 125.1, 124.5, 123.0, 122.8, 122.6, 122.54, 122.51, 122.4, 121.8, 120.8, 119.2, 112.3, 108.5, 33.4 ppm. HRMS (ESI) m/z: $[M+H]^+$ calculated for $C_{21}H_{16}N$, 282.12773; found, 282.12784.

9-Butyl-9*H*-dibenzo[*a*,*c*]carbazole(BCZ-*n*Bu). White solid (195 mg, 61% yield). ^1H NMR (400 MHz, $CDCl_3$) δ: 8.92-8.90 (m, 2H), 8.79 (d, *J* = 8.4 Hz, 1H), 8.65 (d, *J* = 8.0 Hz, 1H), 8.52 (d, *J* = 7.6 Hz, 1H), 7.77 (t, *J* = 6.8 Hz, 1H), 7.70-7.69 (m, 2H), 7.64-7.58 (m, 2H), 7.51 (t, *J* = 7.2 Hz, 1H), 7.42 (t, *J* = 7.6 Hz, 1H), 4.78 (t, *J* = 6.8 Hz, 2H), 2.21-2.01 (m, 2H), 1.60-1.55 (m, 2H), 1.06 (t, *J* = 7.2 Hz, 3H) ppm. ^{13}C NMR (101 MHz, $CDCl_3$) δ: 140.6, 133.8, 131.0, 130.1, 127.4, 126.9, 126.5, 125.6, 124.3, 123.8, 123.73, 123.70, 123.6, 123.5, 123.5, 122.6, 122.0, 120.4, 113.8, 109.8, 46.1, 32.2, 20.4, 14.0 ppm. HRMS (ESI) m/z: $[M+H]^+$ calculated for $C_{24}H_{22}N$, 324.17468; found, 324.17487.

9-Benzyl-9*H*-dibenzo[*a*,*c*]carbazole(BCZ-Be). White solid (285 mg, 80% yield). ^1H NMR (400 MHz, $CDCl_3$) δ: 8.95 (d, *J* = 8.0 Hz, 1H), 8.84 (d, *J* = 8.4 Hz, 1H), 8.79 (d, *J* = 8.4 Hz, 1H), 8.72-8.63 (m, 1H), 8.24 (d, *J* = 8.4 Hz, 1H), 7.80 (t, *J* = 7.2 Hz, 1H), 7.64-7.60 (m, 2H), 7.49-7.45 (m, 4H), 7.40-7.30 (m, 5H), 5.94 (s, 2H) ppm. ^{13}C NMR (101 MHz, $CDCl_3$) δ: 141.3, 137.5, 134.7, 131.0, 130.0, 129.2, 127.6, 127.5, 127.2, 126.5, 126.1, 125.8, 124.2, 124.1, 123.92, 123.90, 123.7, 123.6, 123.3, 122.9, 122.0, 121.0, 114.0, 110.0, 50.2 ppm. HRMS (ESI) m/z: $[M+H]^+$ calculated for $C_{27}H_{20}N$, 358.16012; found, 358.15924.

9-Phenyl-9*H*-dibenzo[*a*,*c*]carbazole (BCZ-Ph). White solid (193 mg, 58% yield). ^1H NMR (500 MHz, $CDCl_3$) δ: 8.84 (d, *J* = 8.0 Hz, 1H), 8.69-8.68 (m, 2H), 8.56 (d, *J* = 7.5 Hz, 1H), 7.69 (t, *J* = 7.0 Hz, 1H), 7.54-7.51 (m, 4H), 7.46-7.32 (m, 5H), 7.29-7.26 (m, 1H), 7.16 (t, *J* = 7.5 Hz, 1H), 7.12-7.10 (m, 1H) ppm. ^{13}C NMR (126 MHz, $CDCl_3$) δ: 142.2, 140.3, 134.6, 130.9, 130.3, 130.0, 129.2, 128.9, 127.44, 127.41, 126.0, 125.8, 124.1, 124.0, 123.89, 123.87, 123.6, 123.3, 121.8, 121.1, 114.3, 111.0 ppm. HRMS (ESI) m/z: $[M+H]^+$ calculated for $C_{26}H_{18}N$, 344.14338; found, 344.14352.

4-(9*H*-Dibenzo[*a*,*c*]carbazol-9-yl)-*N*,*N*-diphenylaniline (BCZ-TPA). White solid (265 mg, 52% yield). ^1H NMR (400 MHz, $CDCl_3$) δ: 8.96 (d, *J* = 8.0 Hz, 1H), 8.83 (t, *J* = 8.0 Hz, 2H), 8.70-8.67 (m, 1H), 7.81 (t, *J* = 7.2 Hz, 1H), 7.75 (d, *J* = 8.4 Hz, 1H), 7.66-7.60 (m, 2H), 7.50-7.43 (m, 3H), 7.41-7.29 (m, 13H), 7.14 (t, *J* = 7.2 Hz, 2H) ppm. ^{13}C NMR (101 MHz, $CDCl_3$) δ: 148.4, 147.4, 142.3, 134.7, 133.4, 130.1, 130.0, 129.8,

129.7, 127.4, 127.3, 125.9, 125.8, 125.1, 123.99, 123.95, 123.9, 123.8, 123.7, 123.6, 123.4, 123.3, 121.7, 121.0, 114.1, 111.1 ppm. HRMS (ESI) m/z: $[M+H]^+$ calculated for $C_{38}H_{27}N_2$, 511.21688; found, 511.21732.

7*H*-Benzo[*c*]carbazole (cBCZ). White solid. ^1H NMR (400 MHz, $CDCl_3$) δ：8.83 (d, *J* = 8.4 Hz, 1H), 8.61 (d, *J* = 8.0 Hz, 1H), 8.19 (br, 1H), 8.05 (d, *J* = 8.4 Hz, 1H), 7.86 (d, *J* = 8.8 Hz, 1H), 7.78-7.74 (m, 1H), 7.56-7.42 (m, 5H) ppm. ^{13}C NMR (126 MHz, $CDCl_3$). δ: 138.5, 137.1, 130.0, 129.3, 127.5, 127.0, 124.4, 124.0, 123.3, 123.1, 122.1, 120.3, 115.4, 112.7, 111.2 ppm. HRMS (ESI) m/z: $[M+H]^+$ calculated for $C_{16}H_{12}N$, 218.09643; found, 218.09610.

7-Methyl-7*H*-benzo[*c*]carbazole (cBCZ-Me). White solid (201 mg, 87% yield). ^1H NMR (500 MHz, $CDCl_3$) δ: 8.83 (d, *J* = 8.5 Hz, 1H), 8.62 (d, *J* = 8.0 Hz, 1H), 8.04 (d, *J* = 8.0 Hz, 1H), 7.92 (d, *J* = 9.0 Hz, 1H), 7.76-7.73 (m, 1H), 7.64 (d, *J* = 9.0 Hz, 1H), 7.56-7.49 (m, 3H), 7.44-7.41 (m, 1H), 3.95 (s, 3H) ppm. ^{13}C NMR (126 MHz, $CDCl_3$) δ 139.9, 138.5, 130.0, 129.3, 128.9, 127.2, 126.9, 124.1, 123.4, 123.2, 122.8, 122.1, 119.8, 114.8, 110.6, 109.1, 29.3 ppm. HRMS (ESI) m/z: $[M+H]^+$ calculated for $C_{17}H_{14}N$, 232.11208; found, 232.11218.

7*H*-Dibenzo[*c,g*]carbazole (cgBCZ). White solid. ^1H NMR (500 MHz, $CDCl_3$) δ: 9.27 (d, *J* = 8.5 Hz, 2H), 8.48 (br, 1H), 8.07 (d, *J* = 8.0 Hz, 2H), 7.85 (d, *J* = 8.5 Hz, 2H), 7.74 (t, *J* = 7.5 Hz, 2H), 7.57 (t, *J* = 7.5 Hz, 2H), 7.51 (d, *J* = 8.5 Hz, 2H).^{13}C NMR (126 MHz, $CDCl_3$) δ: 136.2, 130.0, 129.27, 129.25, 126.8, 125.5, 125.2, 123.4, 117.7, 112.7 ppm. HRMS (ESI) m/z: $[M+H]^+$ calculated for $C_{20}H_{14}N$, 268.11208; found, 268.11136.

7*H*-Dibenzo[*c,g*]carbazole (cgBCZ-Me). White solid (253 mg, 90% yield). ^1H NMR (400 MHz, $CDCl_3$) δ: 9.29-9.25 (m, 2H), 8.10-8.06 (m, 2H), 7.93-7.90 (m, 2H), 7.74-7.66 (m, 4H), 7.58-7.55 (m, 2H), 4.03-4.01 (m, 3H). ^{13}C NMR (126 MHz, $CDCl_3$) δ: 136.2, 130.0, 129.3, 129.5, 126.8, 125.5, 125.2, 123.4, 117.7, 112.7 ppm. ^{13}C NMR (126 MHz, $CDCl_3$) δ: 137.6, 129.7, 129.2, 126.6, 125.4, 125.1, 123.2, 117.1, 110.6, 100.0, 29.4 ppm. HRMS (ESI) m/z: $[M+H]^+$ calculated for $C_{21}H_{16}N$, 282.12773; found, 282.12762.

Supplementary Figures

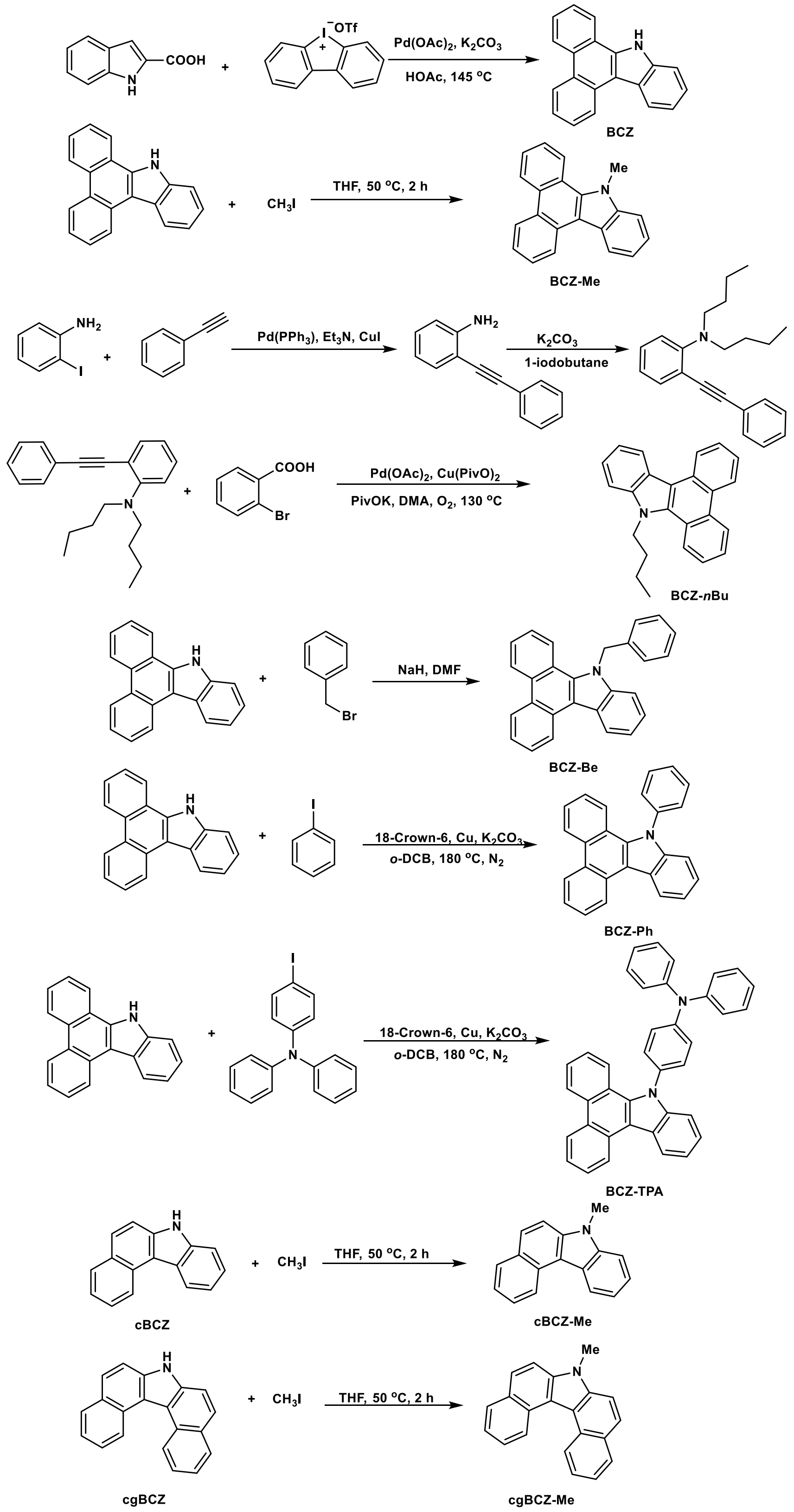

Supplementary Fig. 1. | Synthetic routes of guests. Synthetic routes of guests BCZ, BCZ-Me, BCZ-*n*Bu, BCZ-Be, BCZ-Ph, BCZ-TPA, cBCZ-Me, and cgBCZ-Me [1-4].

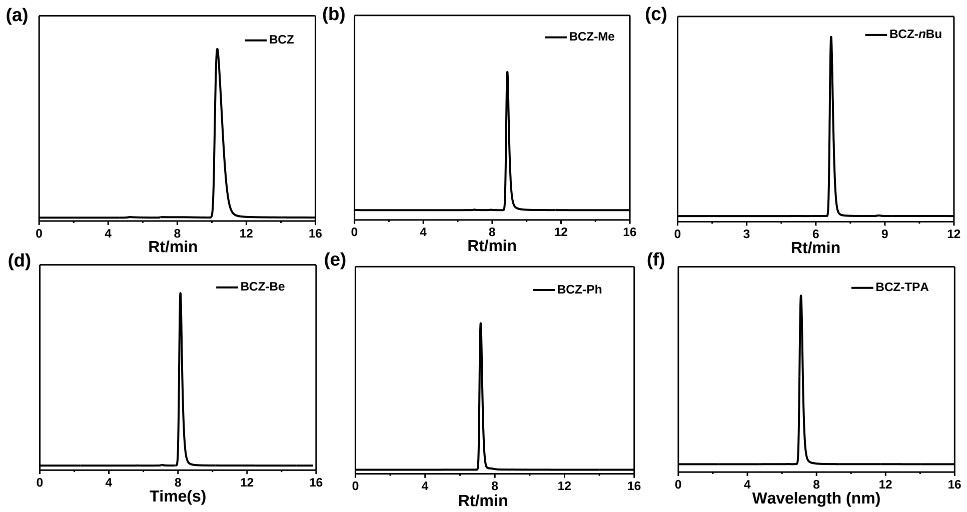

Supplementary Fig. 2. | HPLC spectra. HPLC spectra of six guests (a) BCZ, (b) BCZ–Me, (c) BCZ–*n*Bu, (d) BCZ–Be, (e) BCZ–Ph, and (f) BCZ–TPA (CH_3OH/hexane = 70%: 30%).

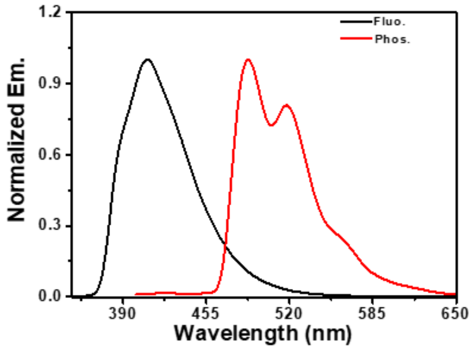

Supplementary Fig. 3. | Emission spectra. Emission spectra of guest BCZ (THF as solvent, 1.0×10^{-5} mol/L, Fluo. at 293 K, Phos. at 77 K, Ex. of Fluo.: 340 nm, Ex. of Phos.: 380 nm).

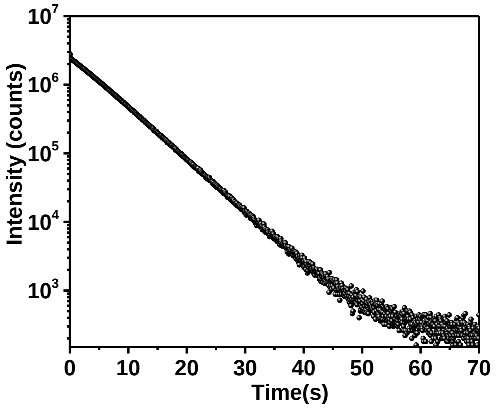

Supplementary Fig. 4. | Kinetic attenuation curve. Kinetic attenuation curve of guest BCZ at 77 K (THF as solvent, 1.0×10^{-5} mol/L, Ex.: 380 nm).

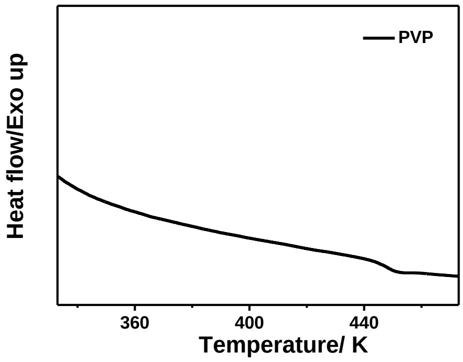

Supplementary Fig. 5. | DSC curve. DSC curve of host PVP.

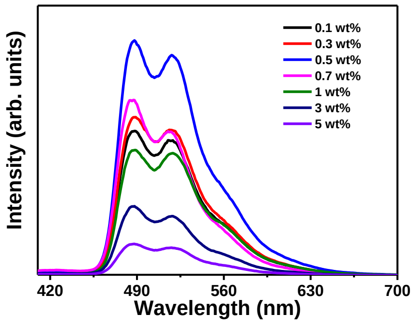

Supplementary Fig. 6. | Emission spectra. Delayed emission spectra of seven doped materials BCZ/PVP with different amounts of BCZ (Delayed time: 1 ms, Ex.: 380 nm).

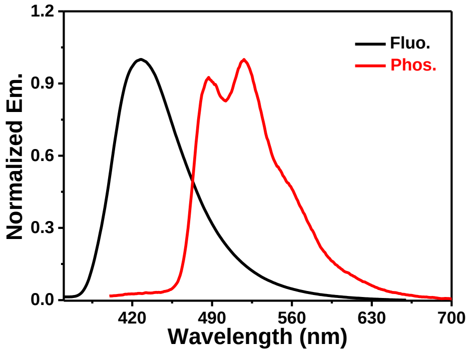

Supplementary Fig. 7. | Emission spectra. Emission spectra of doped material BCZ/PVP (Delayed time: 1 ms, Ex. of Fluo.: 340 nm, Ex. of Phos.: 380 nm).

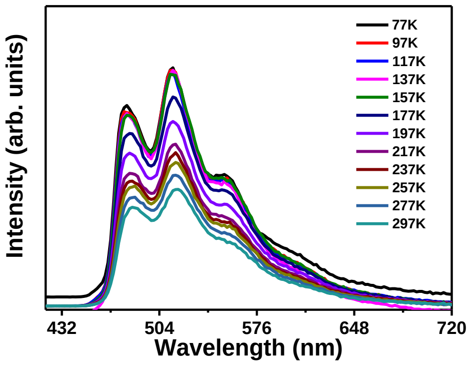

Supplementary Fig. 8. | Emission spectra. Delayed emission spectra of doped material BCZ/PVP at different temperatures (Delayed time: 1 ms, Ex.: 380 nm).

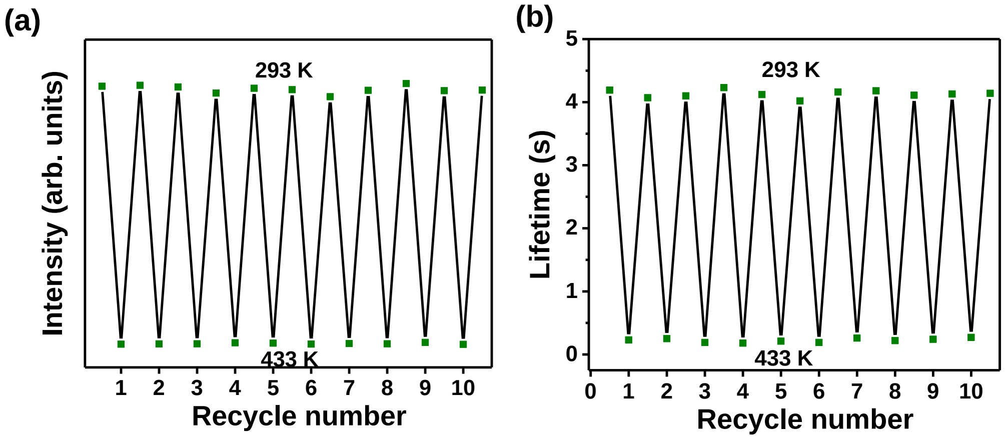

Supplementary Fig. 9. | Cycles spectra. (a) Phosphorescence intensity and (b) phosphorescence lifetime of doped material BCZ/PVP under temperature rise and fall cycles (Delayed time: 1 ms, Ex.: 380 nm).

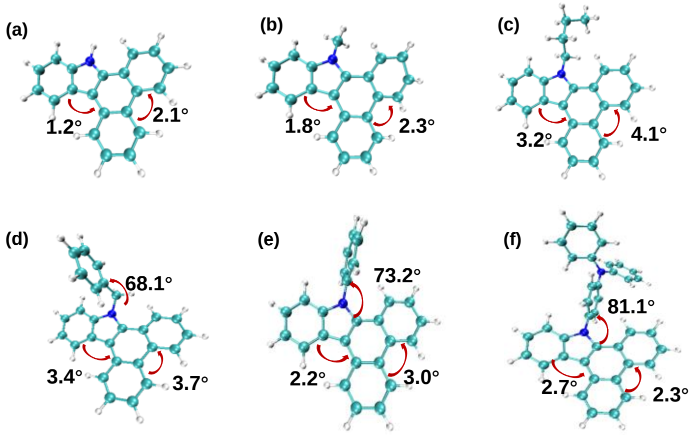

Supplementary Fig. 10. | Molecular configuration. Theoretical molecular configuration of six guests (a) BCZ, (b) BCZ–Me, (c) BCZ–*n*Bu, (d) BCZ–Be, (e) BCZ–Ph, and (f) BCZ–TPA.

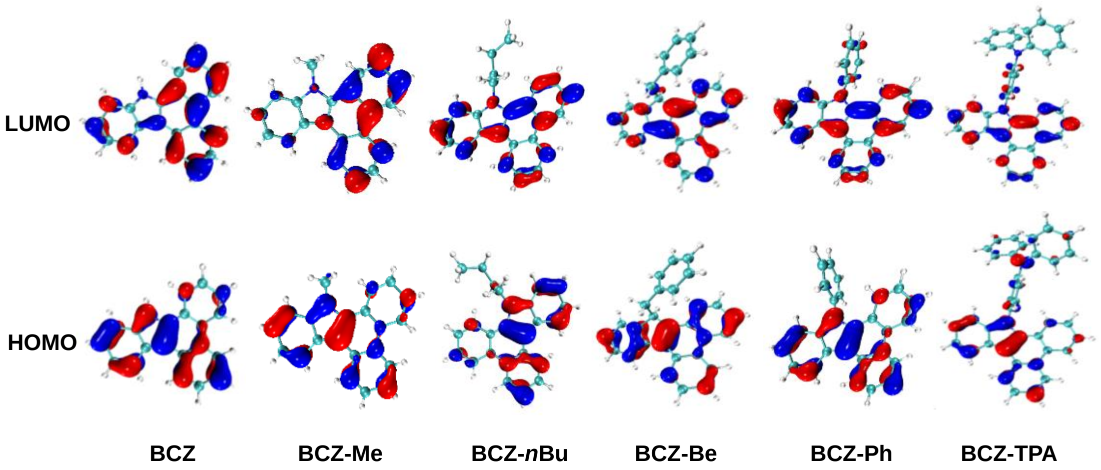

Supplementary Fig. 11. | LUMO and HOMO distribution. LUMO and HOMO distribution images of six guests.

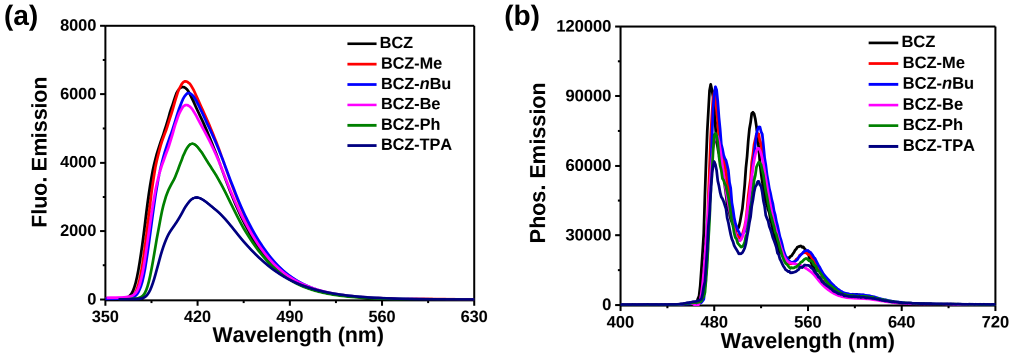

Supplementary Fig. 12. | Emission spectra. (a) Fluorescence emission spectra of six guests, (b) Phosphorescence emission spectra of six guests (THF as solvent, 1.0×10^{-5} mol/L, Fluo. at 293 K, Phos. at 77 K, Ex. of Fluo.: 340 nm, Ex. of Phos.: 380 nm).

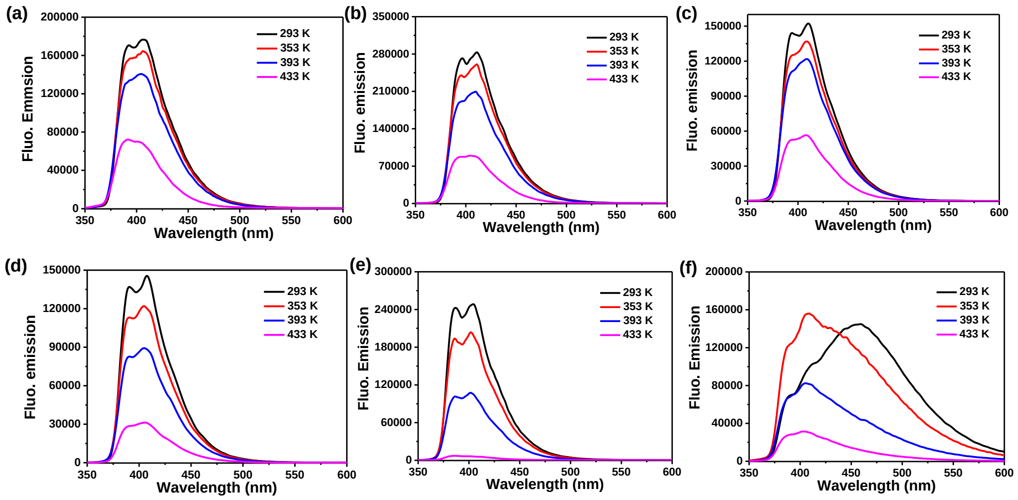

Supplementary Fig. 13. | Emission spectra. Fluorescence emission spectra of six guests (a) BCZ, (b) BCZ–Me, (c) BCZ–*n*Bu, (d) BCZ–Be, (e) BCZ–Ph, and (f) BCZ–TPA at different temperatures (DMSO as solvent, 1.0×10^{-5} mol/L, Ex.: 340 nm).

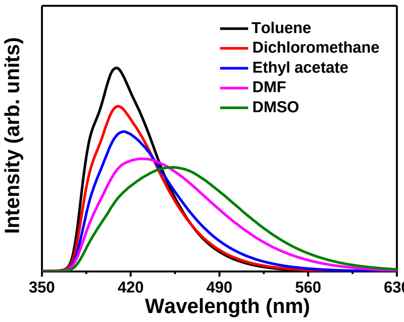

Supplementary Fig. 14. | Emission spectra. Fluorescence emission spectra of BCZ-TPA at different solvent (1.0×10^{-5} mol/L, Ex.: 340 nm).

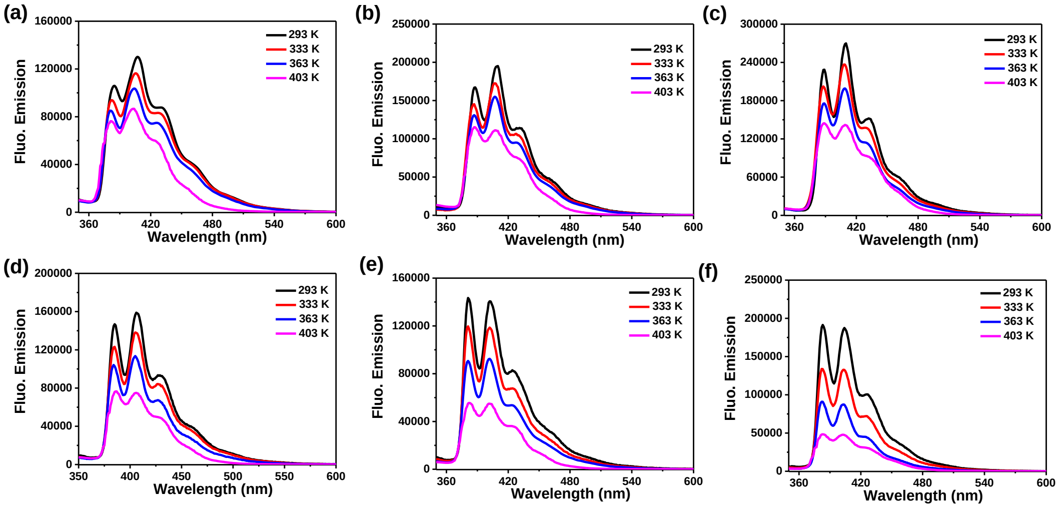

Supplementary Fig. 15. | Emission spectra. Fluorescence emission spectra of six guests (a) BCZ, (b) BCZ–Me, (c) BCZ–*n*Bu, (d) BCZ–Be, (e) BCZ–Ph, and (f) BCZ–TPA at different temperatures (*p*-xylene as solvent, 1.0×10^{-5} mol/L, Ex.: 340 nm).

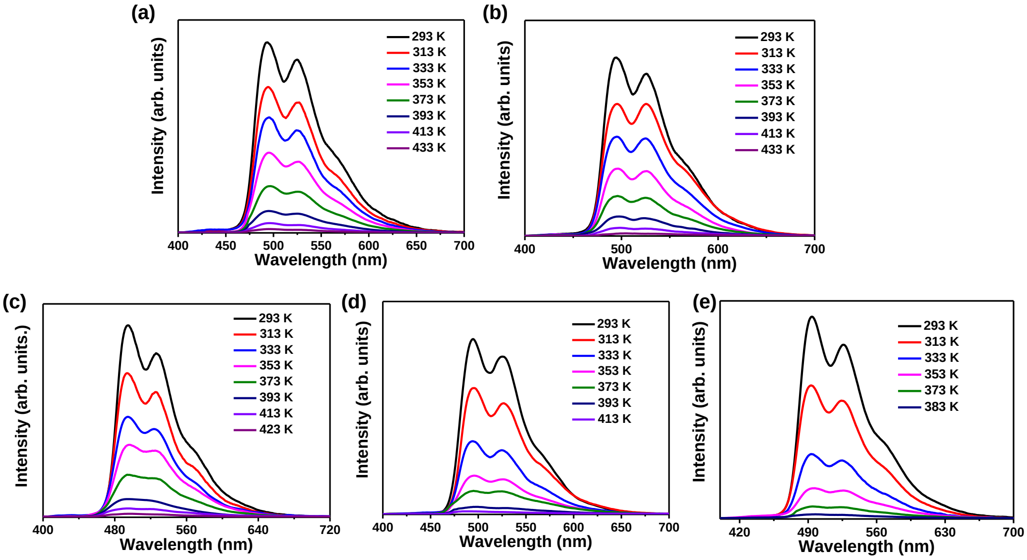

Supplementary Fig. 16. | Emission spectra. Phosphorescence spectra of five doped materials (a) BCZ-Me/PVP, (b) BCZ-*n*Bu/PVP, (c) BCZ-Be/PVP, (d) BCZ-Ph/PVP, and (e) BCZ-TPA/PVP at different temperatures (Delayed time: 1 ms, Ex.: 380 nm).

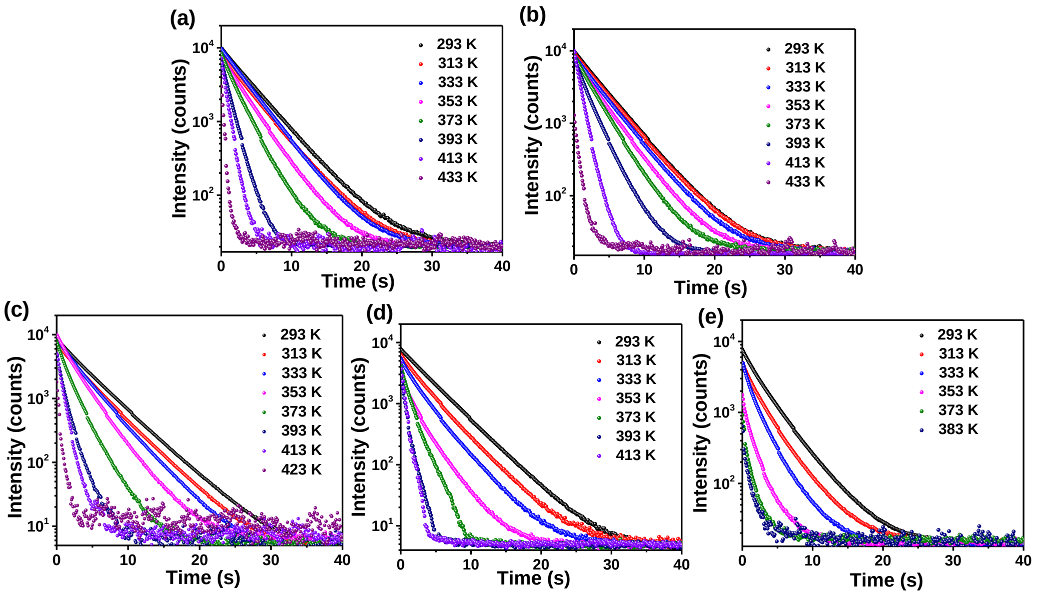

Supplementary Fig. 17. | Kinetic attenuation curves. Kinetic attenuation curves of five doped materials (a) BCZ-Me/PVP, (b) BCZ-*n*Bu/PVP, (c) BCZ-Be/PVP, (d) BCZ-Ph/PVP, and (e) BCZ-TPA/PVP at different temperatures (Ex.: 380 nm).

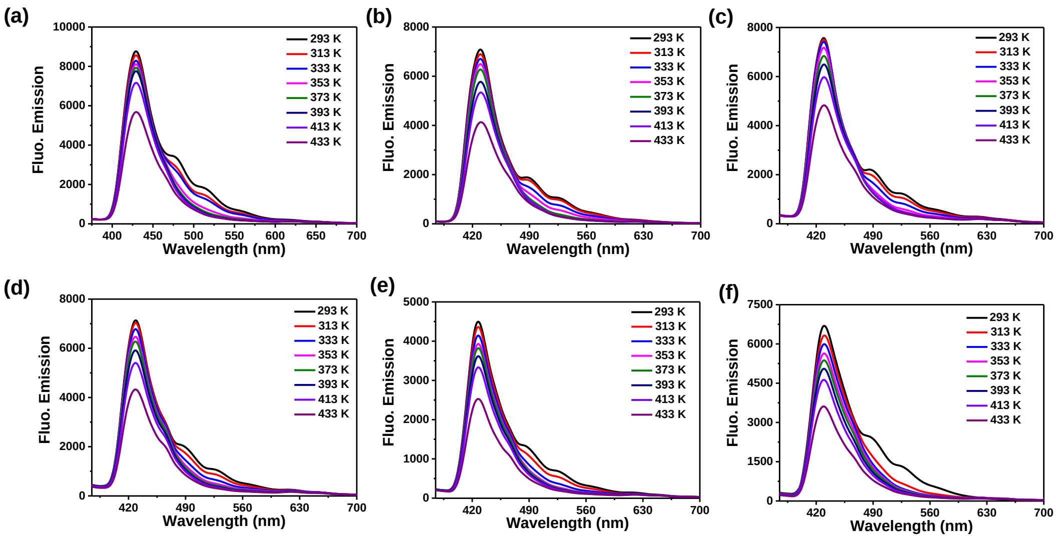

Supplementary Fig. 18. | Emission spectra. Fluorescence emission spectra of six doped materials (a) BCZ/PVP, (b) BCZ-Me/PVP, (c) BCZ-*n*Bu/PVP, (d) BCZ-Be/PVP, (e) BCZ-Ph/PVP, (f) BCZ-TPA/PVP at different temperatures (Ex.: 340 nm).

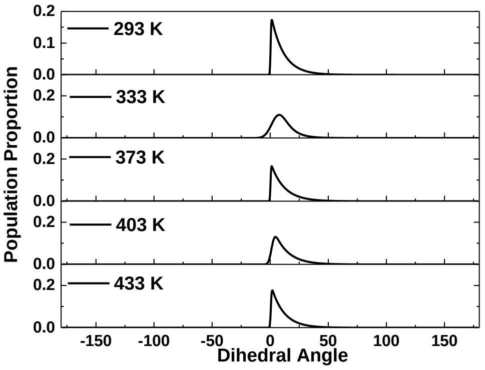

Supplementary Fig. 19. | Distribution spectra. Distribution of the torsion angle at position 2 of BCZ-TPA at different temperatures.

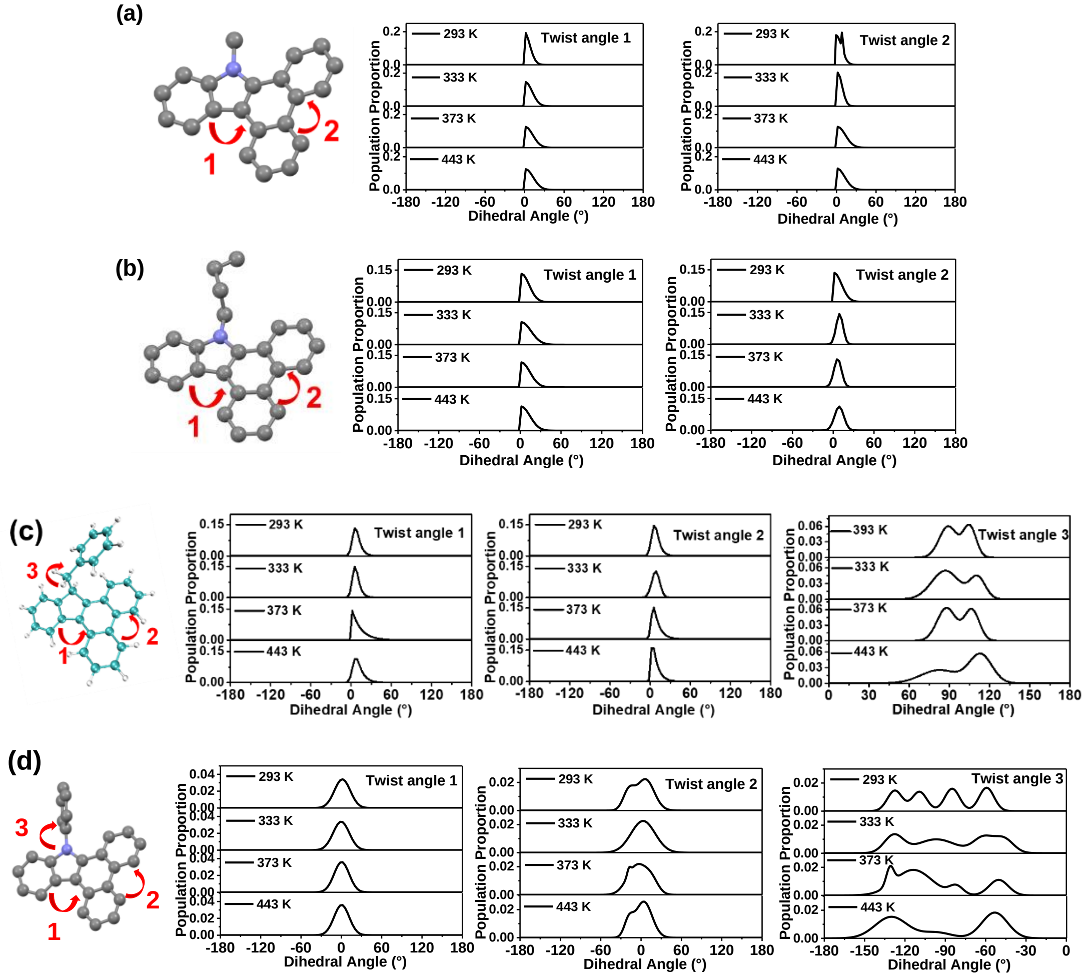

Supplementary Fig. 20. | Distribution spectra. Distribution of the torsion angles of four guests (a) BCZ-Me, (b) BCZ-*n*Bu, (c) BCZ-Be, and (d) BCZ-Ph at different temperatures.

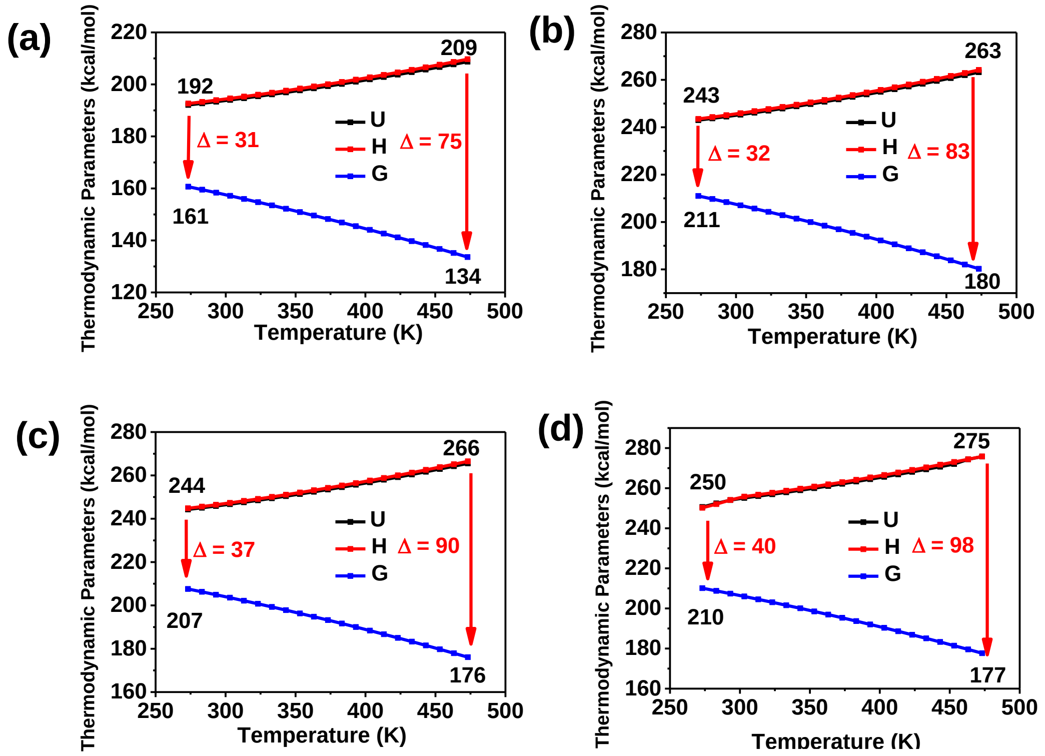

Supplementary Fig. 21. | Thermodynamic parameters. Thermodynamic parameters of four guests (a) BCZ-Me, (b) BCZ-*n*Bu, (c) BCZ-Be, and (d) BCZ-Ph at different temperatures.

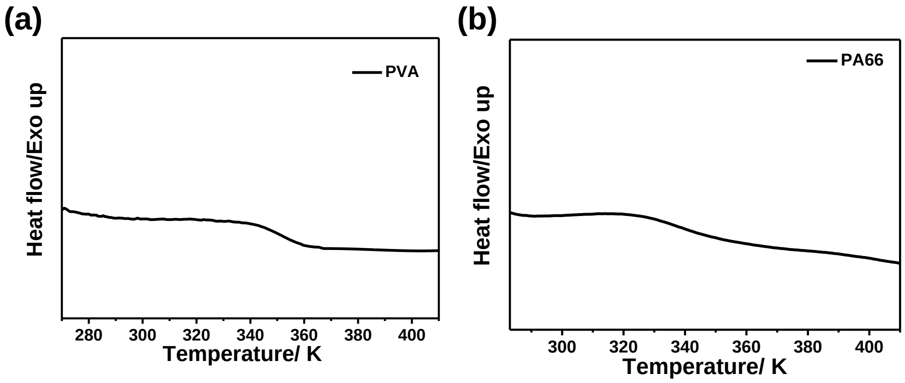

Supplementary Fig. 22. | DSC curves. DSC curves of (a) PVA and (b) PA66.

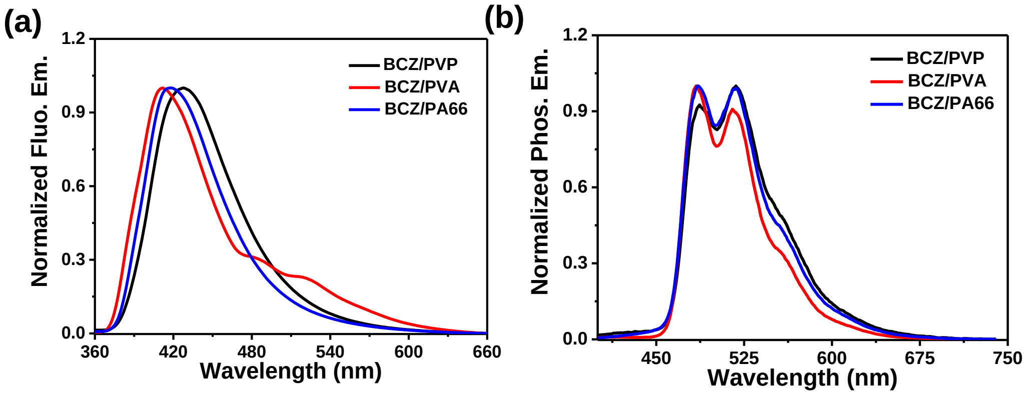

Supplementary Fig. 23. | Emission spectra. (a) Fluorescence emission spectra and (b) phosphorescence emission spectra of three doped materials BCZ/PVP, BCZ/PVA, and BCZ/PA66 (Ex. of Fluo.: 340 nm, Ex. of Phos.: 380 nm).

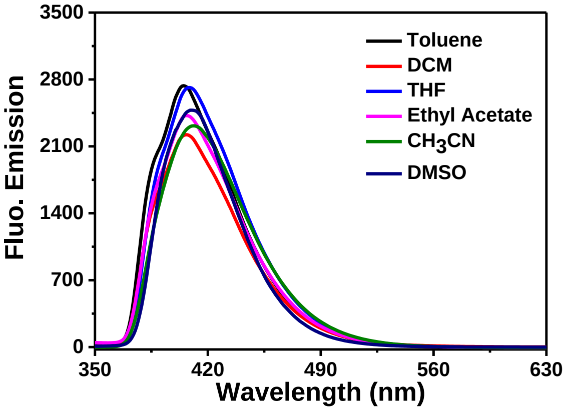

Supplementary Fig. 24. | Emission spectra. Fluorescence emission spectra of BCZ at different solvent (1.0×10^{-5} mol/L, Ex.: 340 nm).

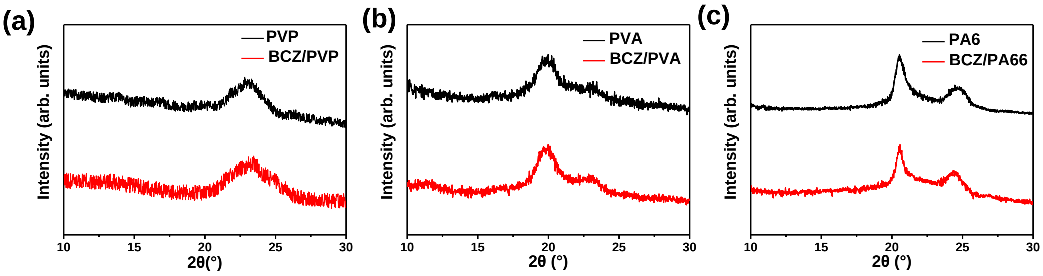

Supplementary Fig. 25. | XRD curves. XRD curves of (a) PVP and BCZ/PVP, (b) PVA and BCZ/PVA, (c) PA66 and BCZ/PA66.

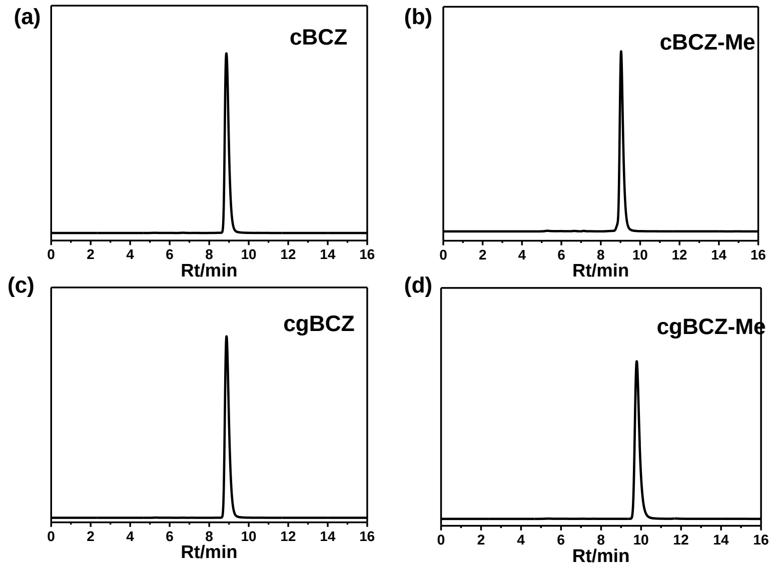

Supplementary Fig. 26. | HPLC spectra. HPLC spectra of (a) cBCZ, (b) cBCZ-Me, (c) cgBCZ, and (d) cgBCZ-Me (CH_3OH/hexane = 70%: 30%).

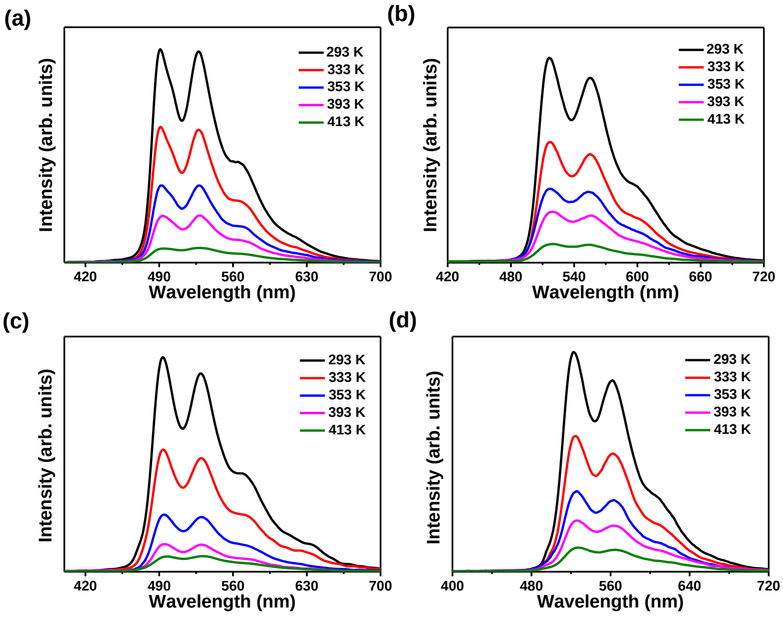

Supplementary Fig. 27. | Emission spectra. Phosphorescence emission spectra of four doped materials (a) cBCZ/PVP, (b) cgBCZ/PVP, (c) cBCZ-Me/PVP, and (d) cgBCZ-Me/PVP at different temperatures (Ex.: 380 nm; Delayed time: 1 ms).

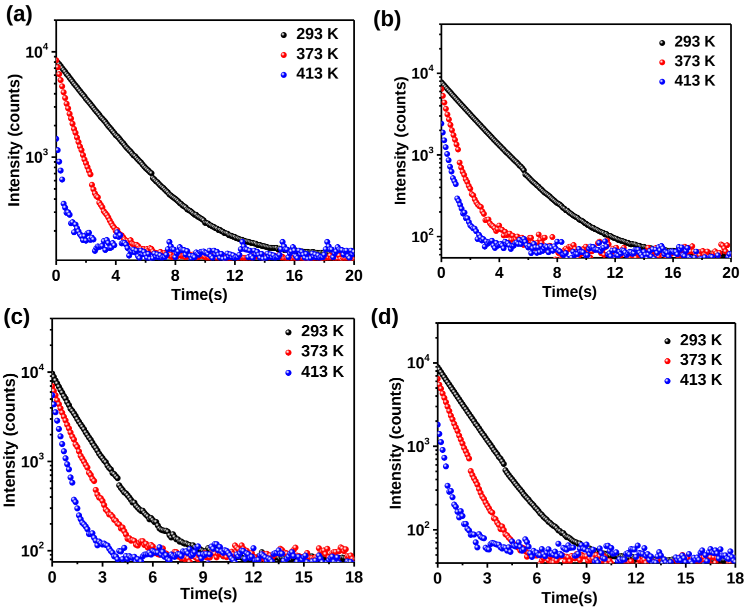

Supplementary Fig. 28. | Kinetic attenuation curves. Kinetic attenuation curves of four doped materials (a) cBCZ/PVP, (b) cgBCZ/PVP, (c) cBCZ-Me/PVP, and (d) cgBCZ-Me/PVP at different temperatures. Ex.: 380 nm.

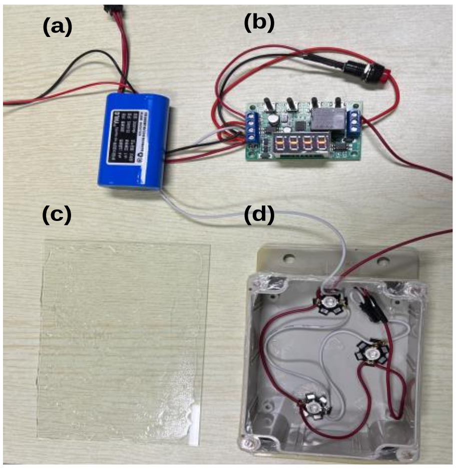

Supplementary Fig. 29. | Device photograph. Device consists of (a) battery, (b) controller, (c) HTP film, and (d) excitation light bulbs.

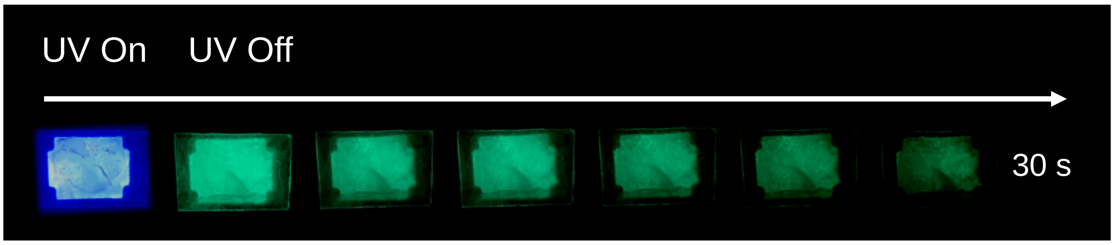

Supplementary Fig. 30. | Luminescence photograph. Luminescence phenomenon of the device in the normal environment.

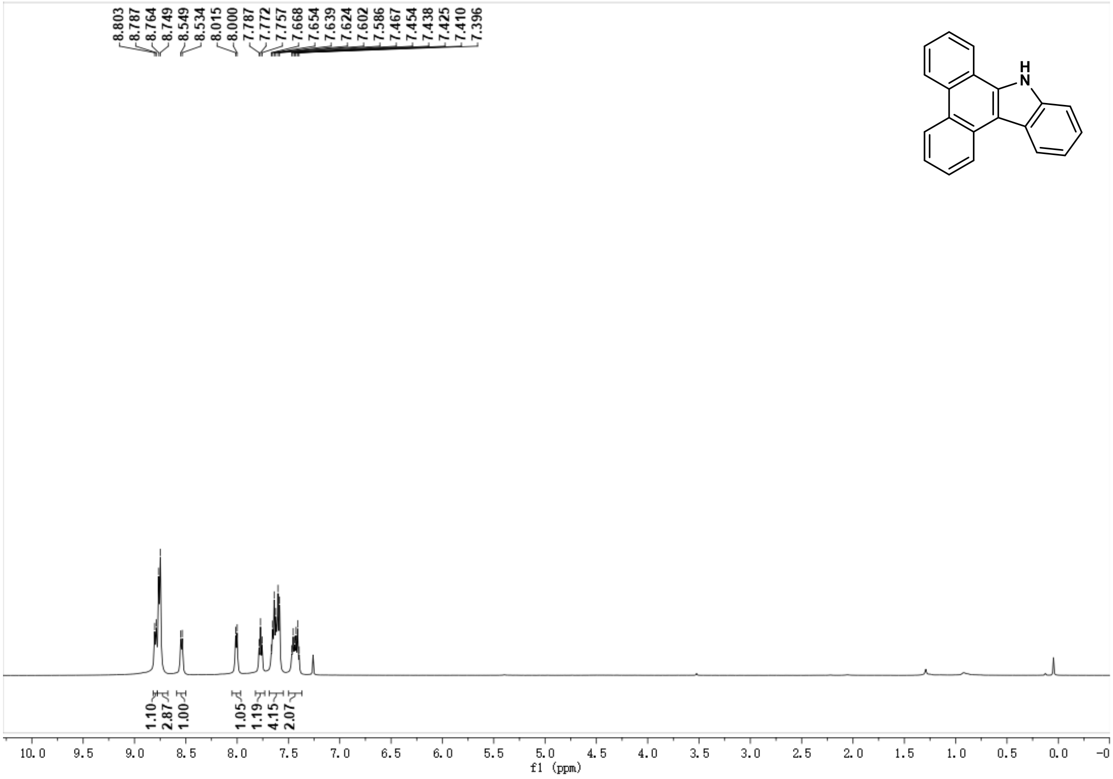

Supplementary Fig. 31. | NMR spectra. ^{1}H NMR of BCZ ($CDCl_3$, 500 MHz).

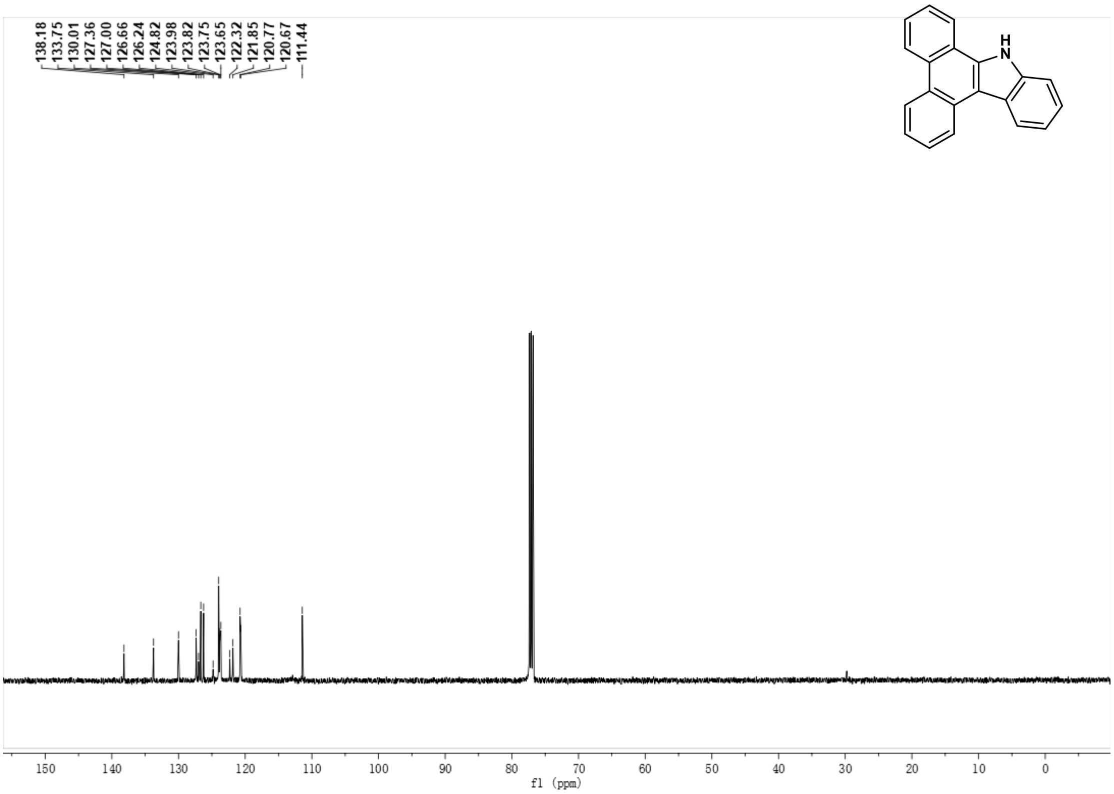

Supplementary Fig. 32. | NMR spectra. ^{13}C NMR of BCZ ($CDCl_3$, 126 MHz).

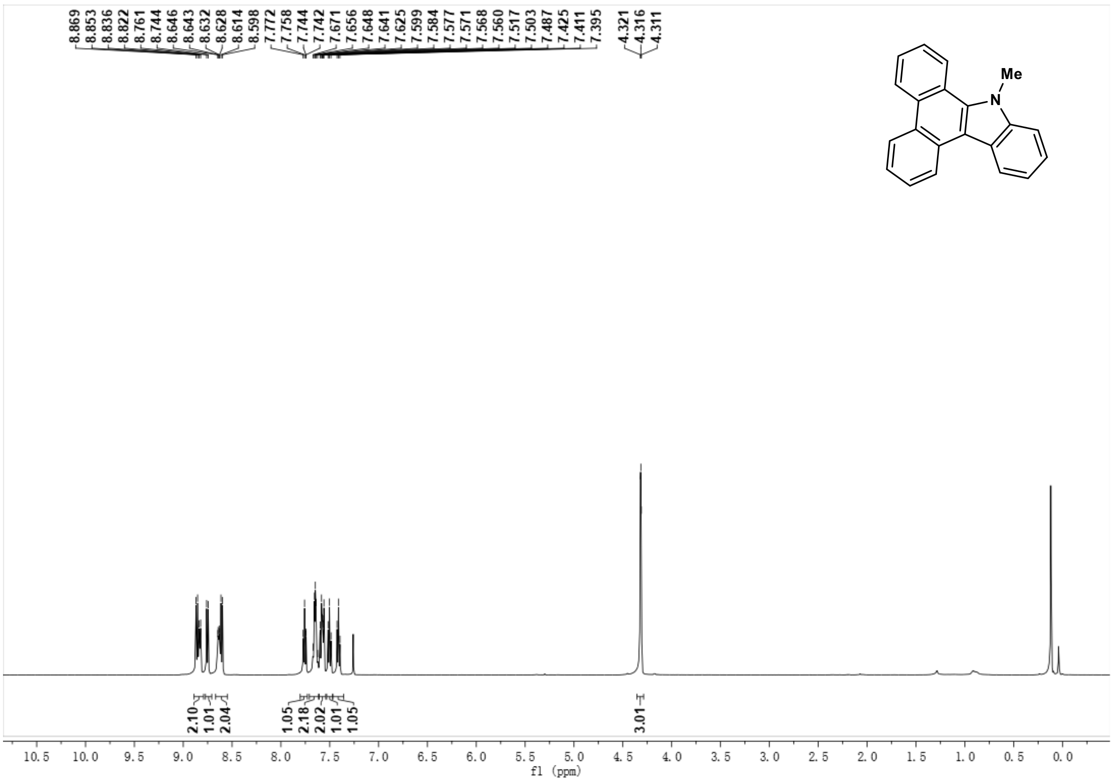

Supplementary Fig. 33. | NMR spectra. ^{1}H NMR of BCZ-Me ($CDCl_3$, 500 MHz).

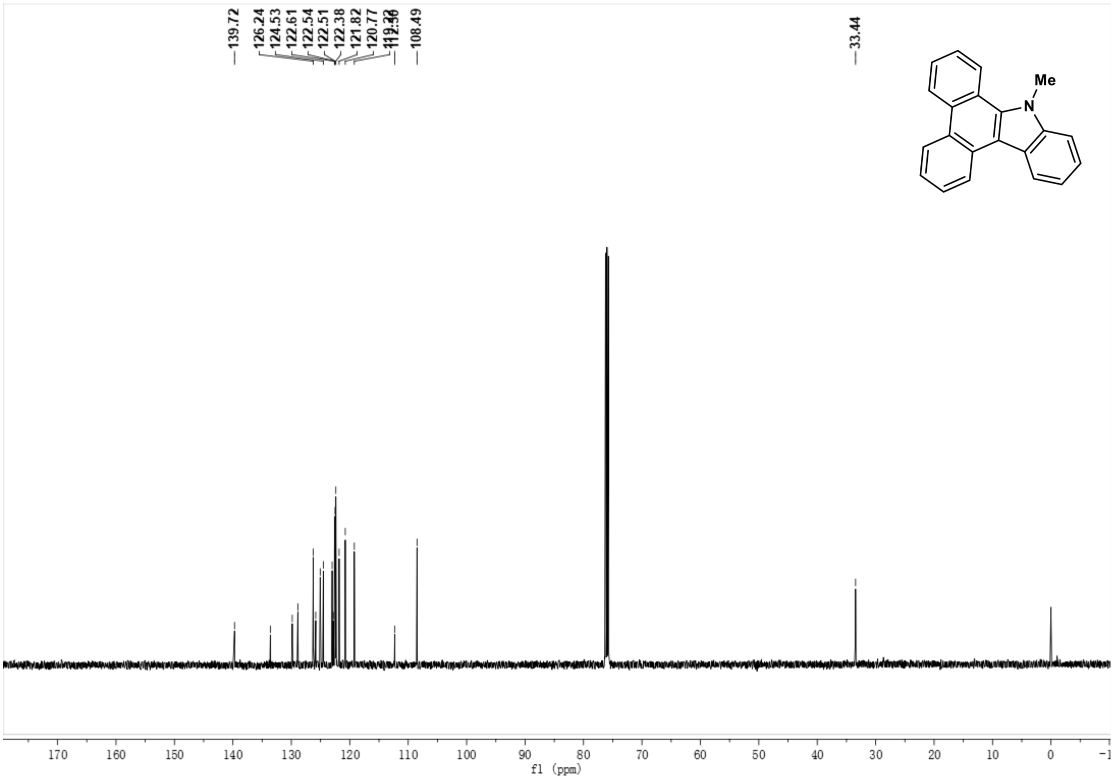

Supplementary Fig. 34. | NMR spectra. ^{13}C NMR of BCZ-Me ($CDCl_3$, 126 MHz).

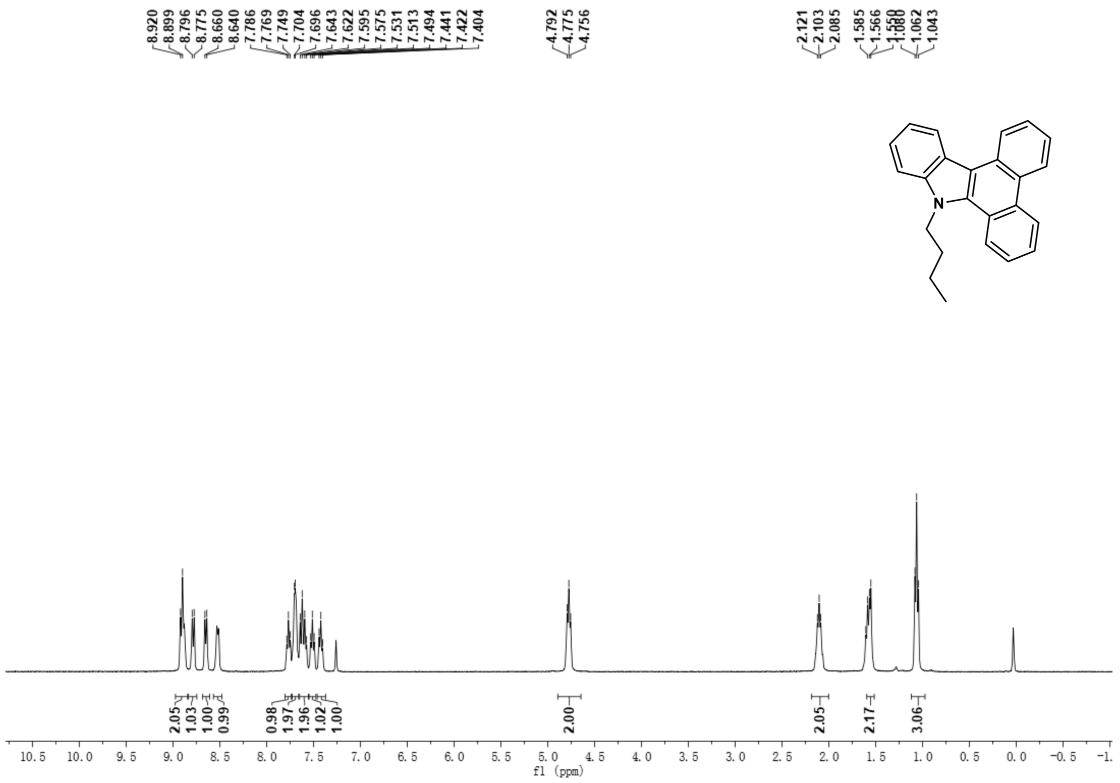

Supplementary Fig. 35. | NMR spectra. ^{1}H NMR of BCZ-*n*Bu ($CDCl_3$, 400 MHz).

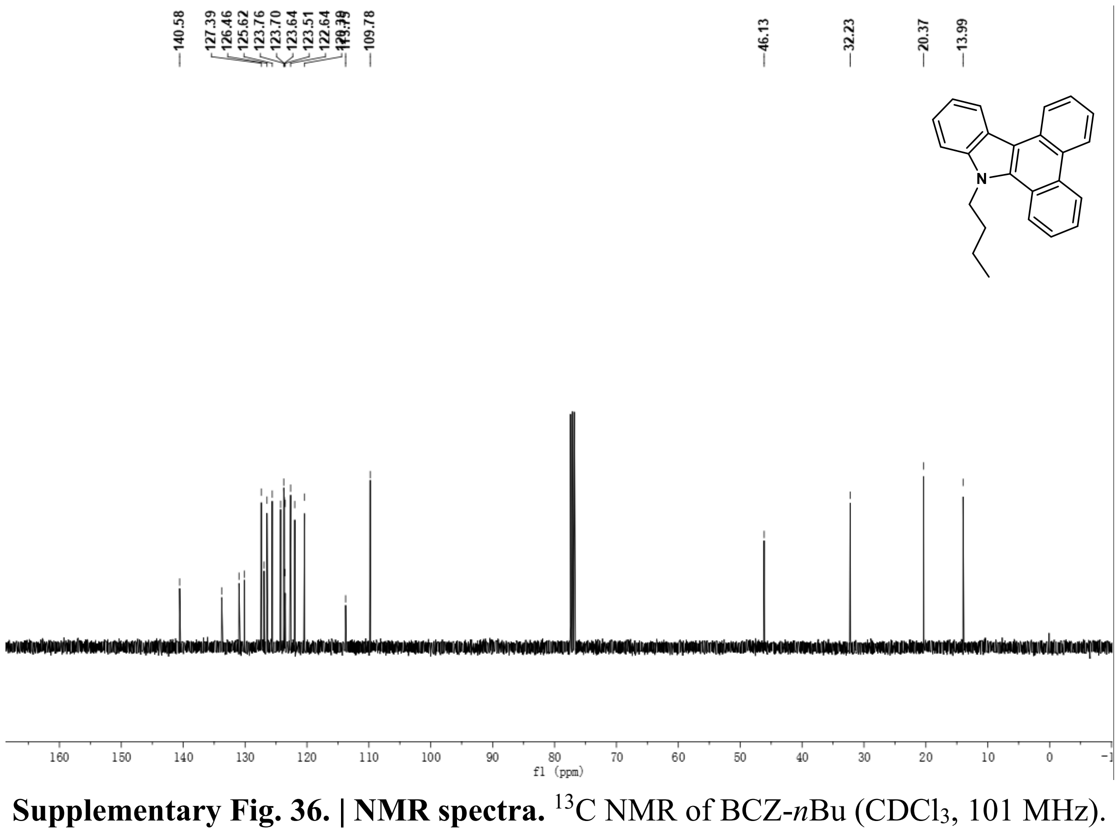

Supplementary Fig. 36. | NMR spectra. ^{13}C NMR of BCZ-*n*Bu ($CDCl_3$, 101 MHz).

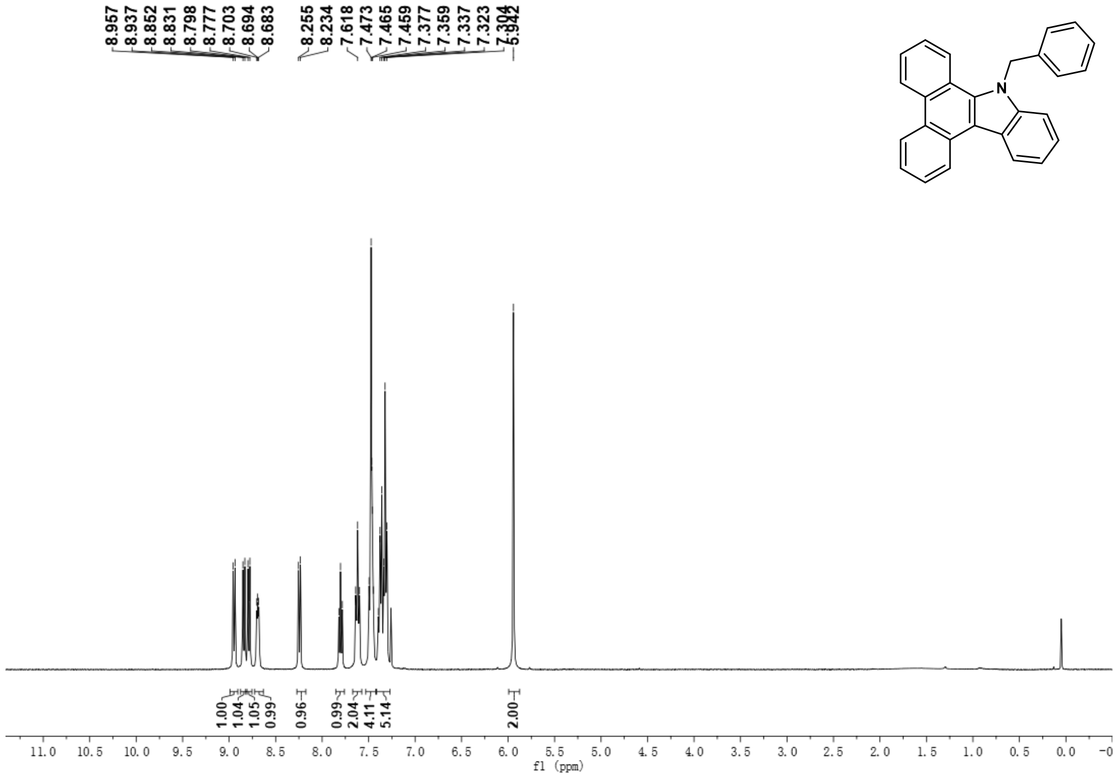

Supplementary Fig. 37. | NMR spectra. ^{1}H NMR of BCZ-Be ($CDCl_3$, 400 MHz).

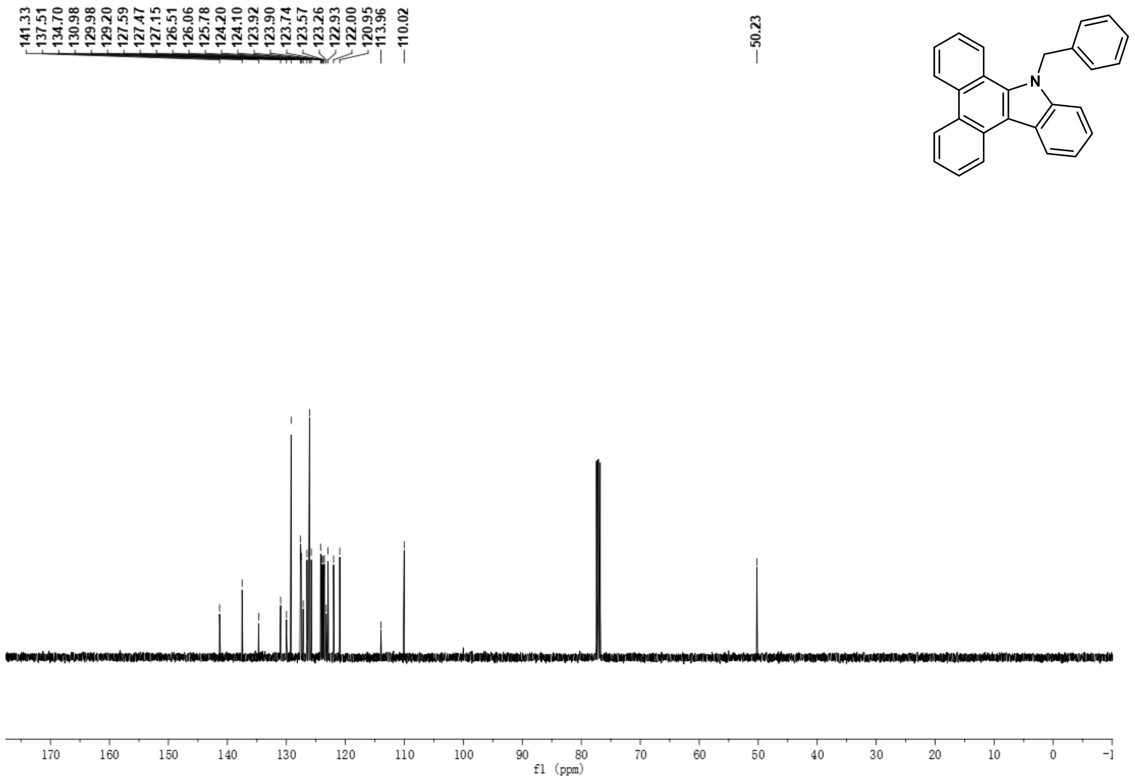

Supplementary Fig. 38. | NMR spectra. ^{13}C NMR of BCZ-Be ($CDCl_3$, 101 MHz).

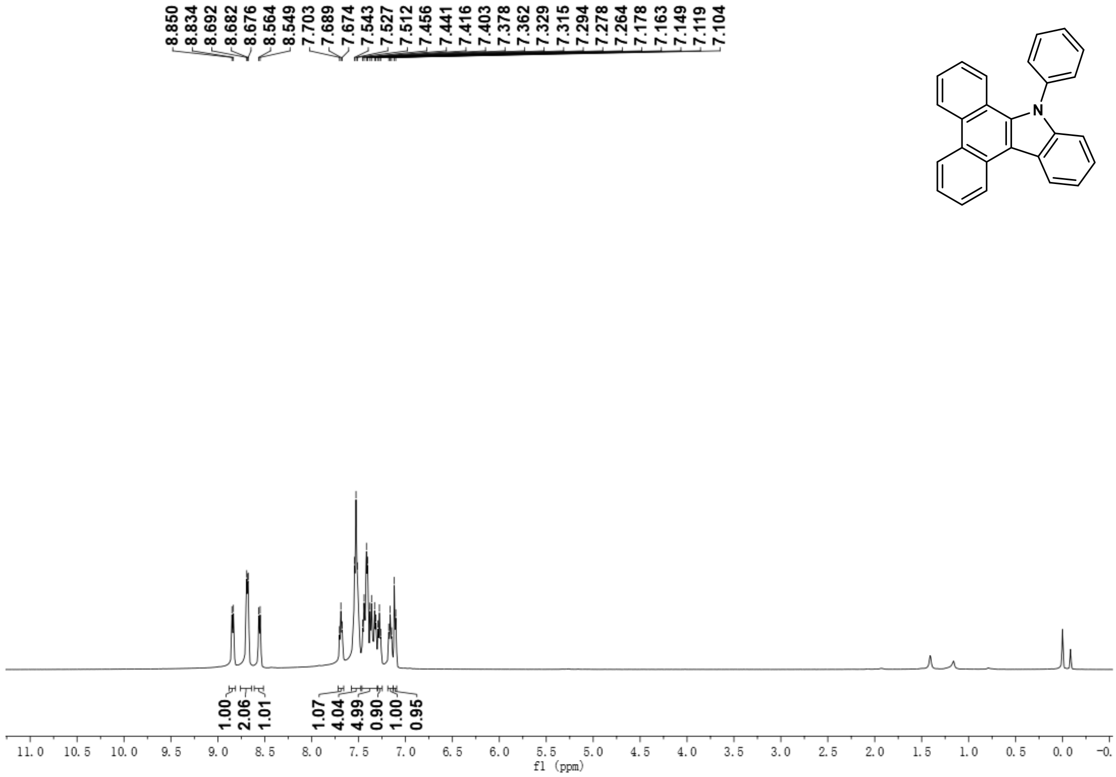

Supplementary Fig. 39. | NMR spectra. ^{1}H NMR of BCZ-Ph ($CDCl_3$, 500 MHz).

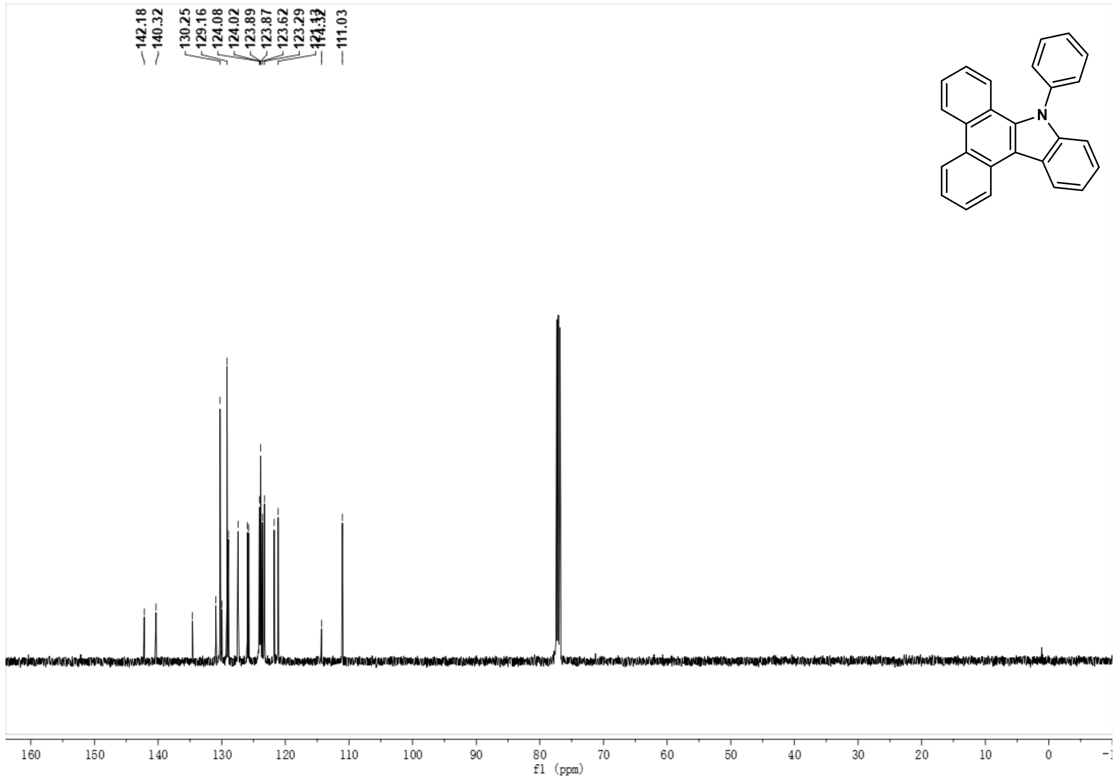

Supplementary Fig. 40. | NMR spectra. ^{13}C NMR of BCZ-Ph ($CDCl_3$, 126 MHz).

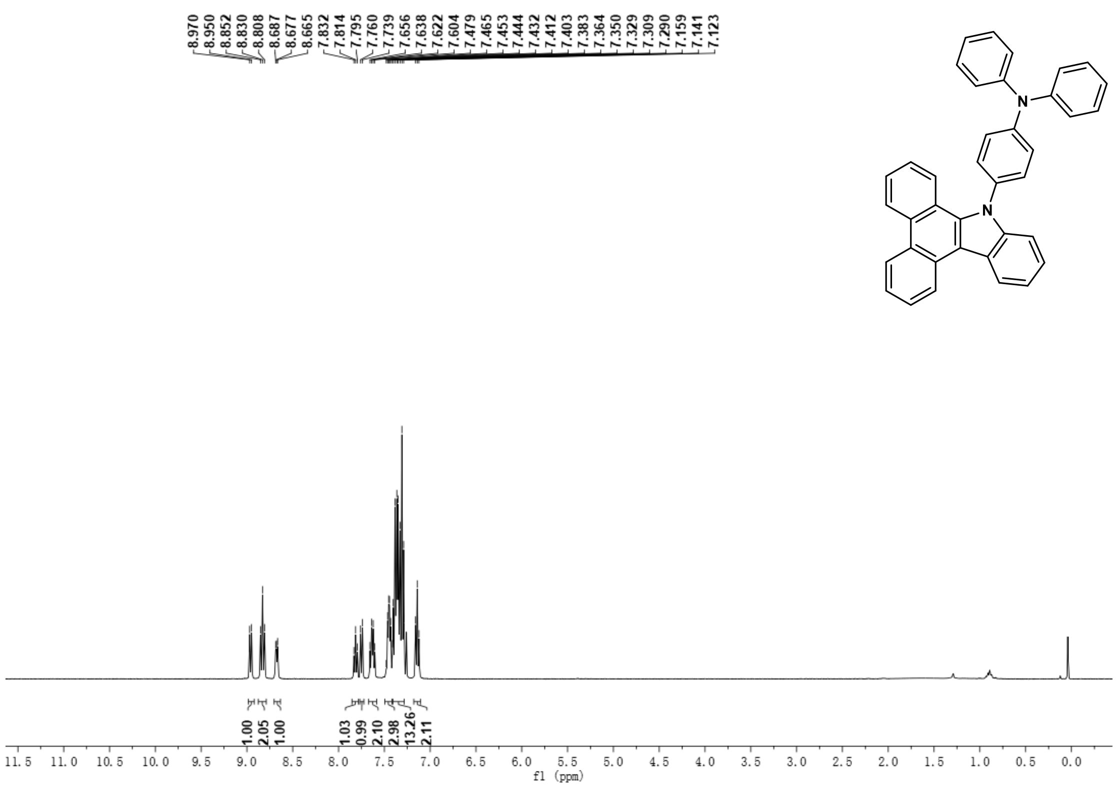

Supplementary Fig. 41. | NMR spectra. ^{1}H NMR of BCZ-TPA ($CDCl_3$, 400 MHz).

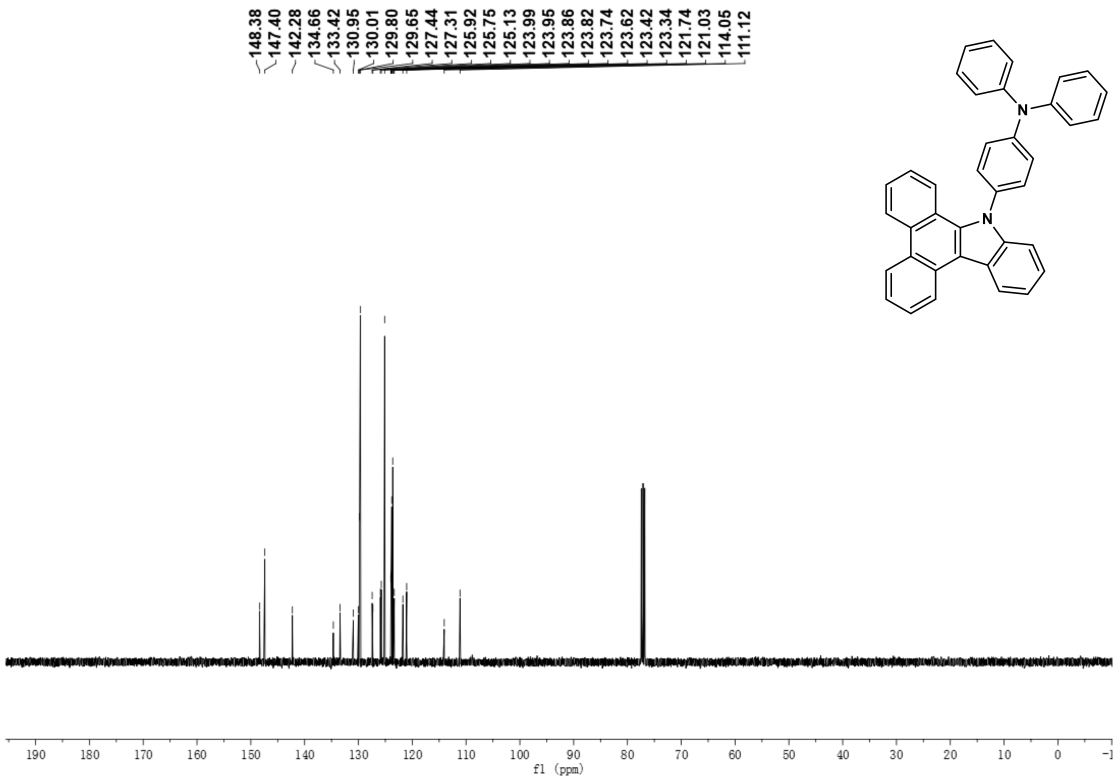

Supplementary Fig. 42. | NMR spectra. ^{13}C NMR of BCZ-TPA ($CDCl_3$, 101 MHz).

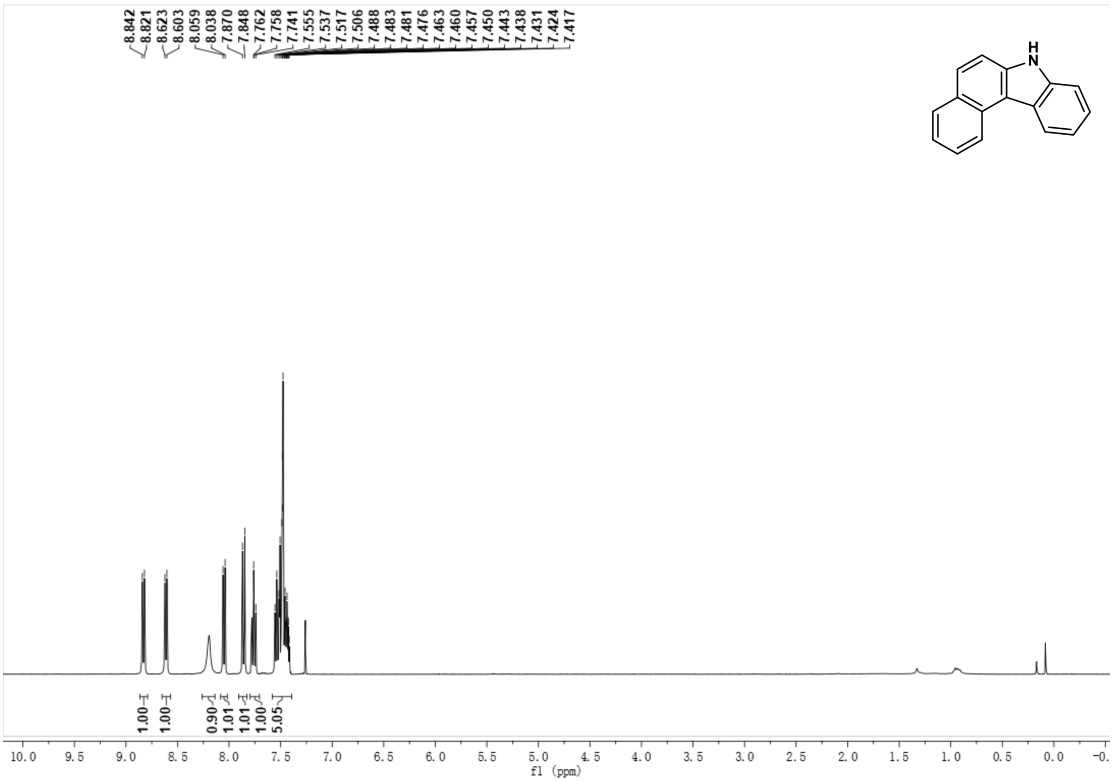

Supplementary Fig. 43. | NMR spectra. ^{1}H NMR of cBCZ ($CDCl_3$, 400 MHz).

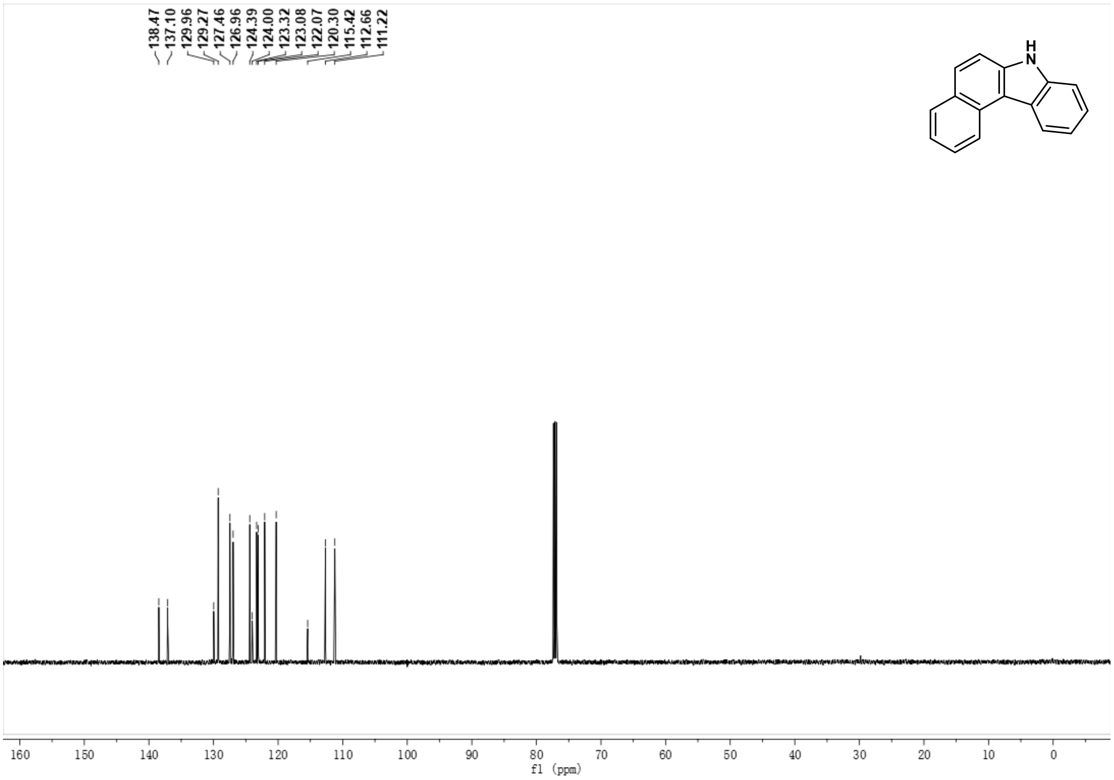

Supplementary Fig. 44. | NMR spectra. ^{13}C NMR of cBCZ ($CDCl_3$, 126 MHz).

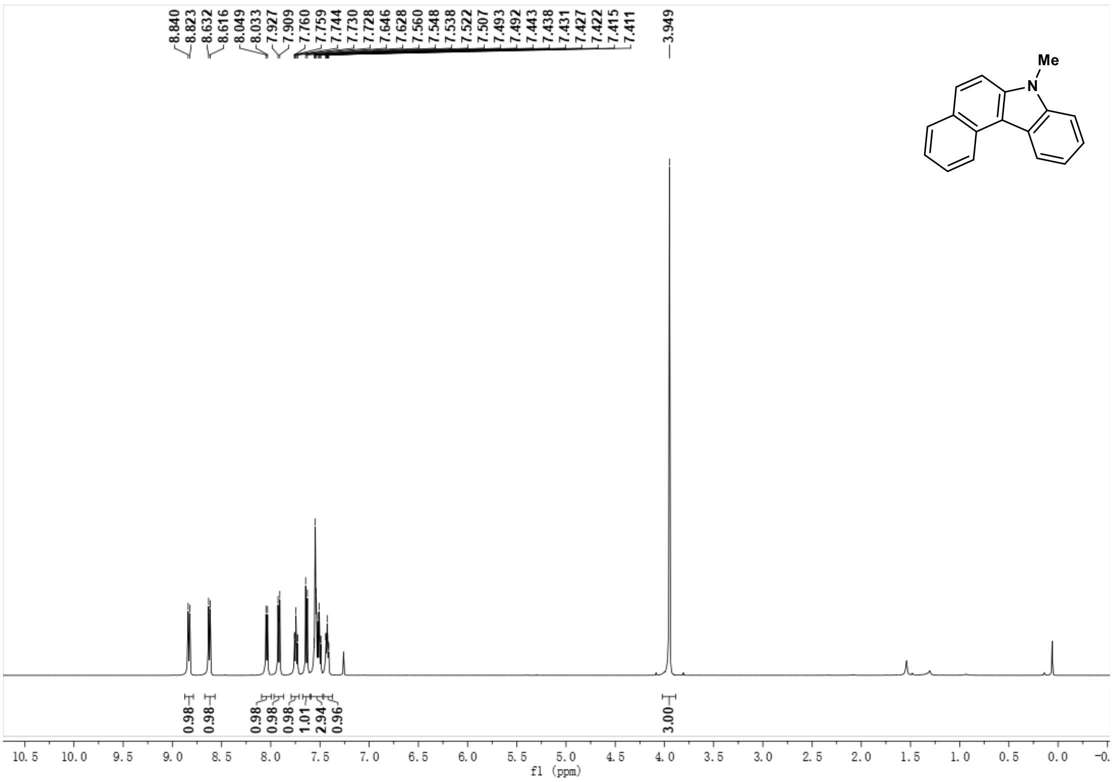

Supplementary Fig. 45. | NMR spectra. ^{1}H NMR of cBCZ-Me ($CDCl_3$, 500 MHz).

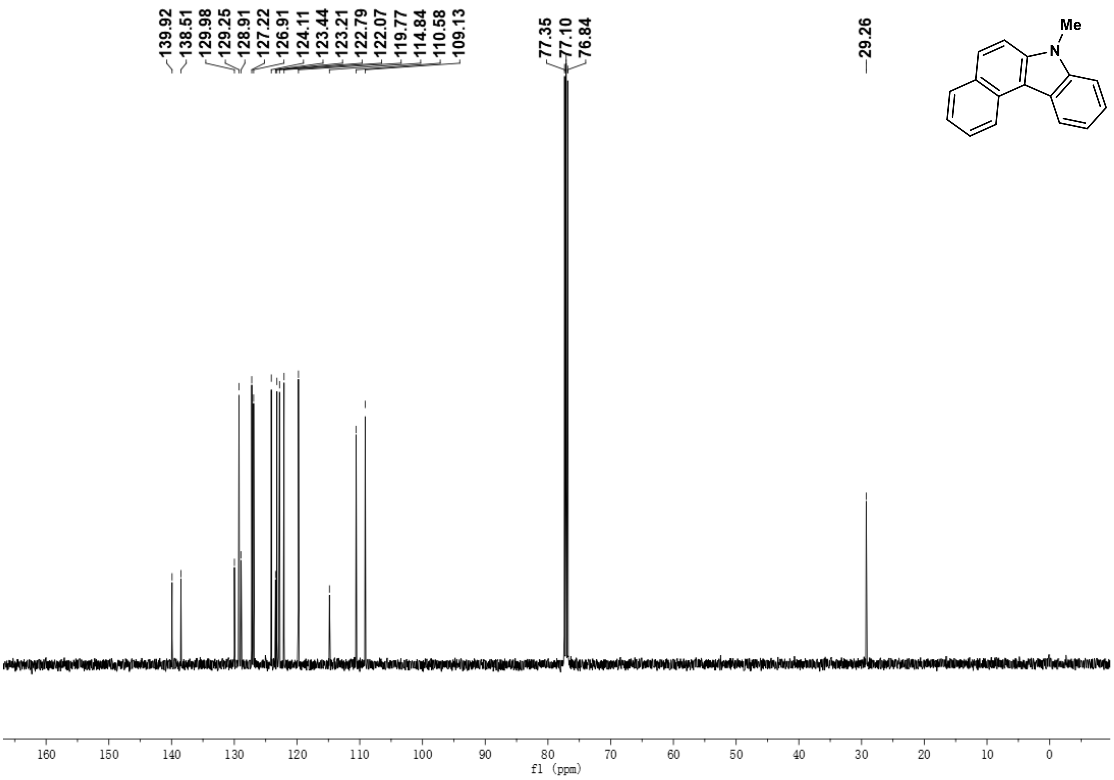

Supplementary Fig. 46. | NMR spectra. ^{13}C NMR of cBCZ-Me ($CDCl_3$, 126 MHz).

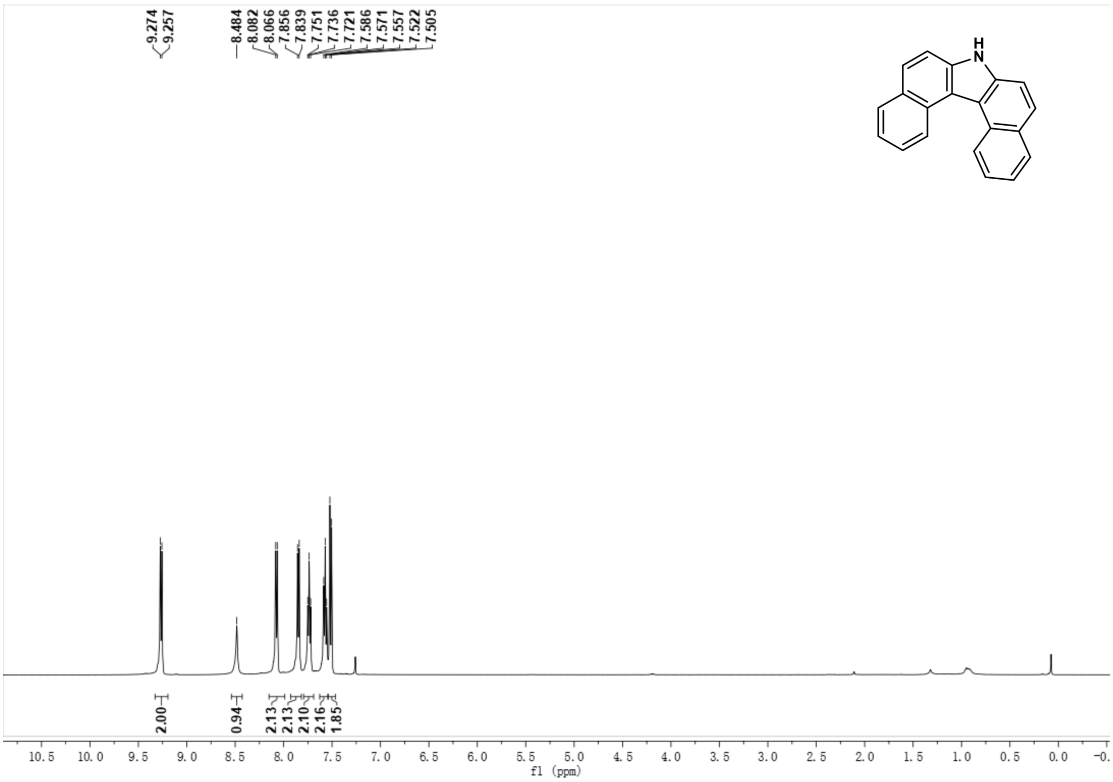

Supplementary Fig. 47. | NMR spectra. ^{1}H NMR of cgBCZ($CDCl_3$, 500 MHz).

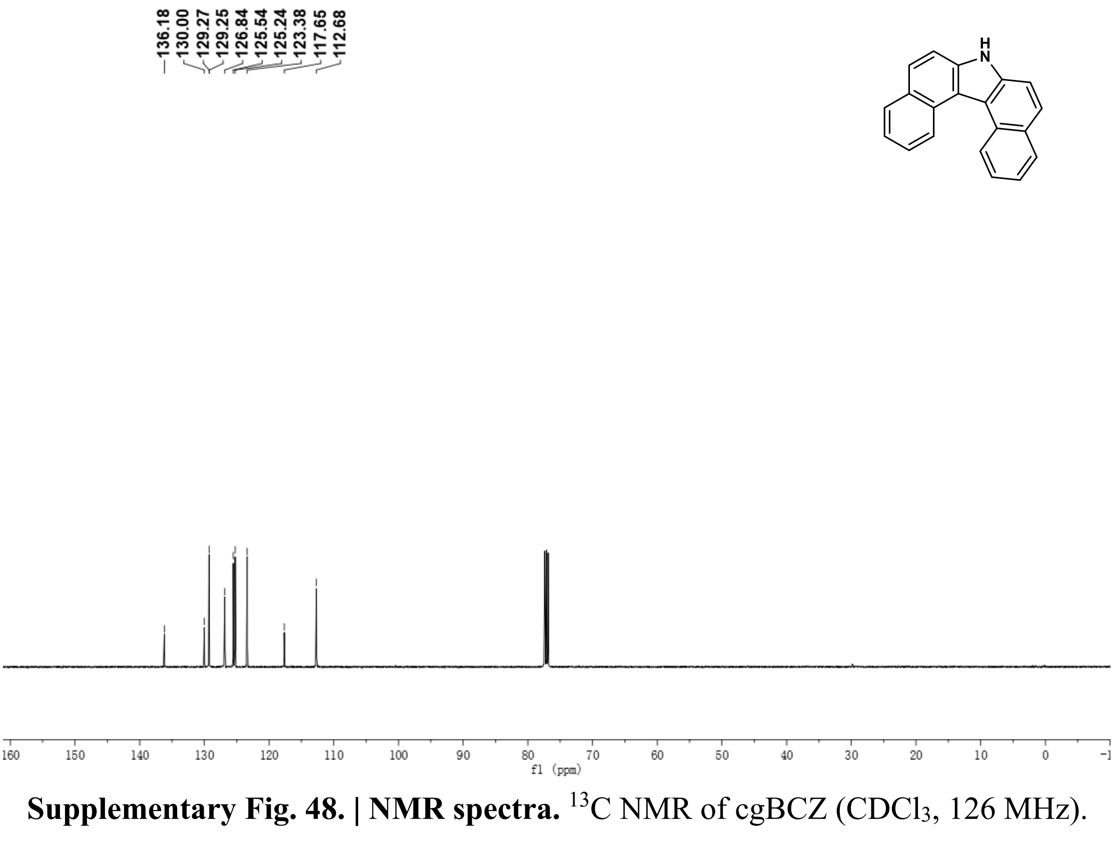

Supplementary Fig. 48. | NMR spectra. ^{13}C NMR of cgBCZ ($CDCl_3$, 126 MHz).

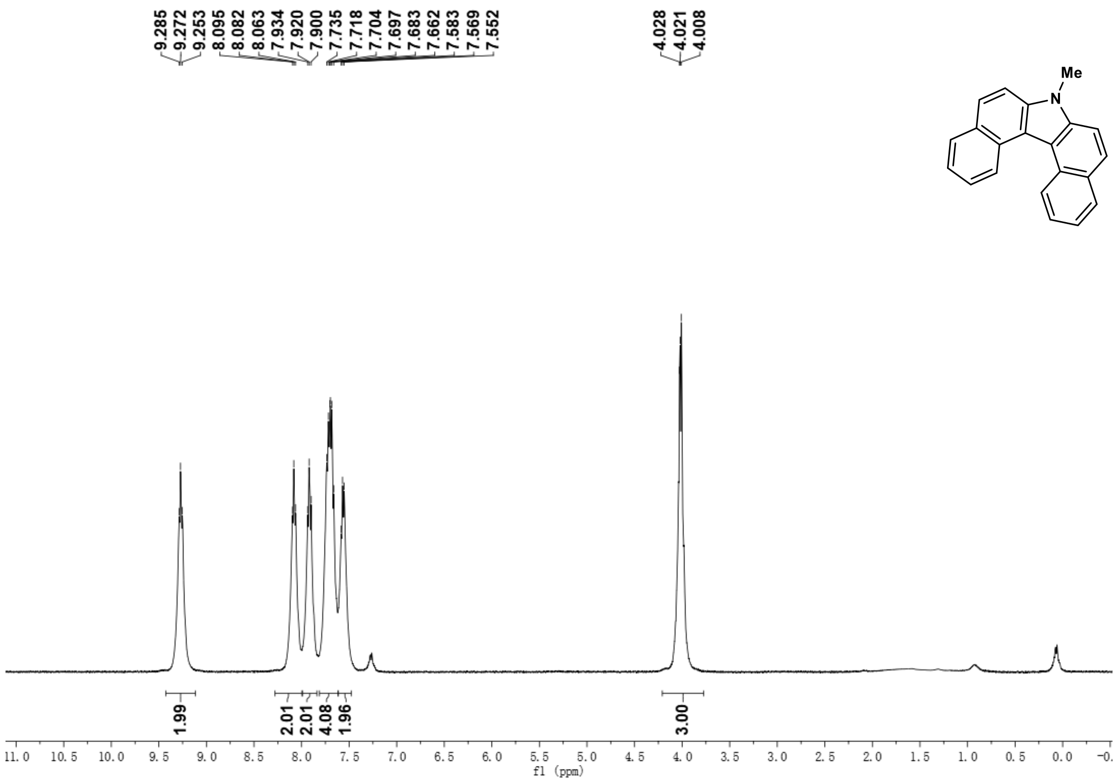

Supplementary Fig. 49. | NMR spectra. ^{1}H NMR of cgBCZ-Me ($CDCl_3$, 400 MHz).

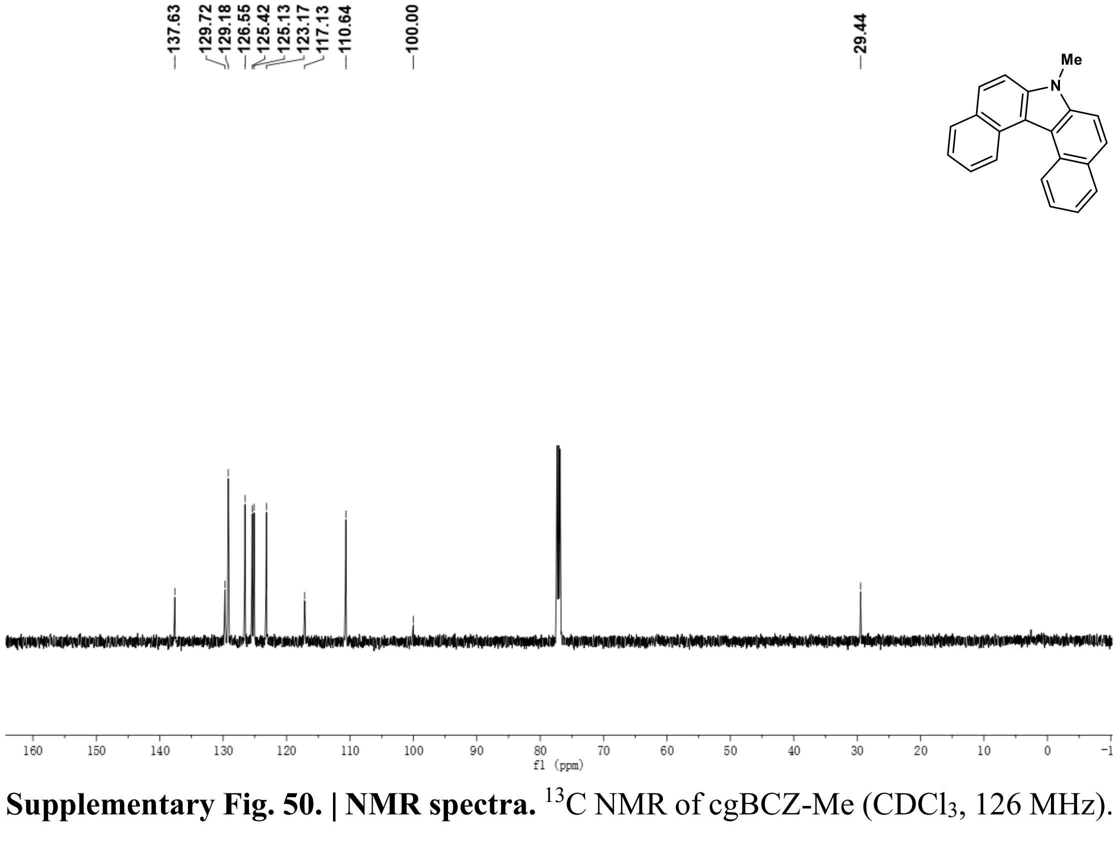

Supplementary Fig. 50. | NMR spectra. ^{13}C NMR of cgBCZ-Me ($CDCl_3$, 126 MHz).

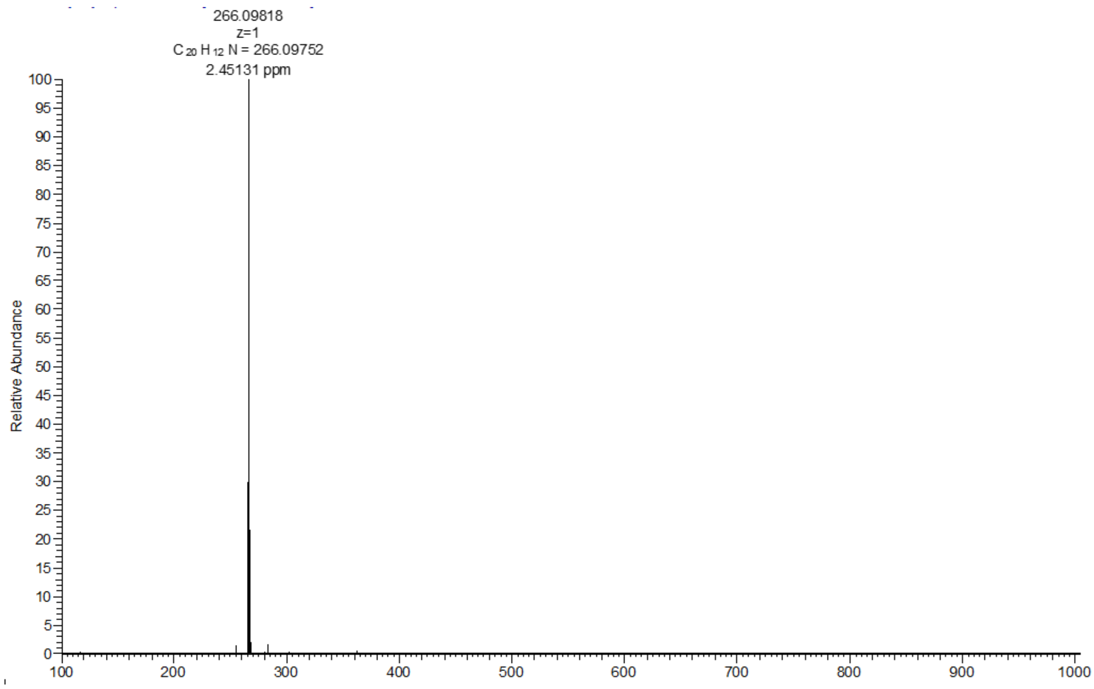

Supplementary Fig. 51. | HRMS spectra. High-resolution mass spectrometry of BCZ.

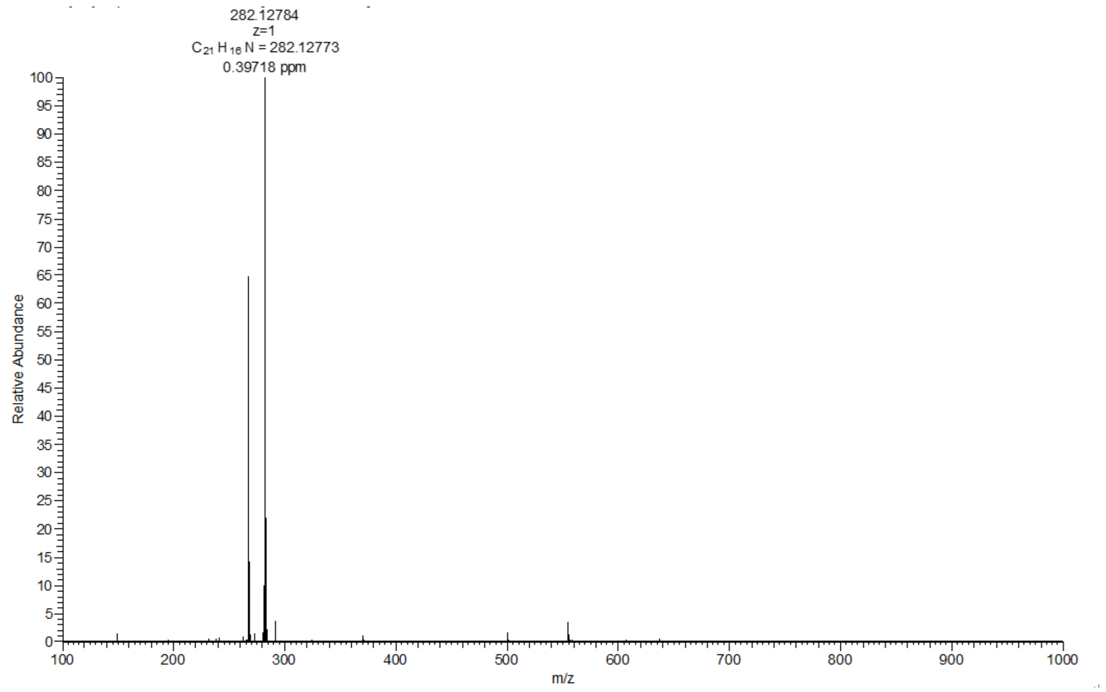

Supplementary Fig. 52. | HRMS spectra. High-resolution mass spectrometry of BCZ-Me.

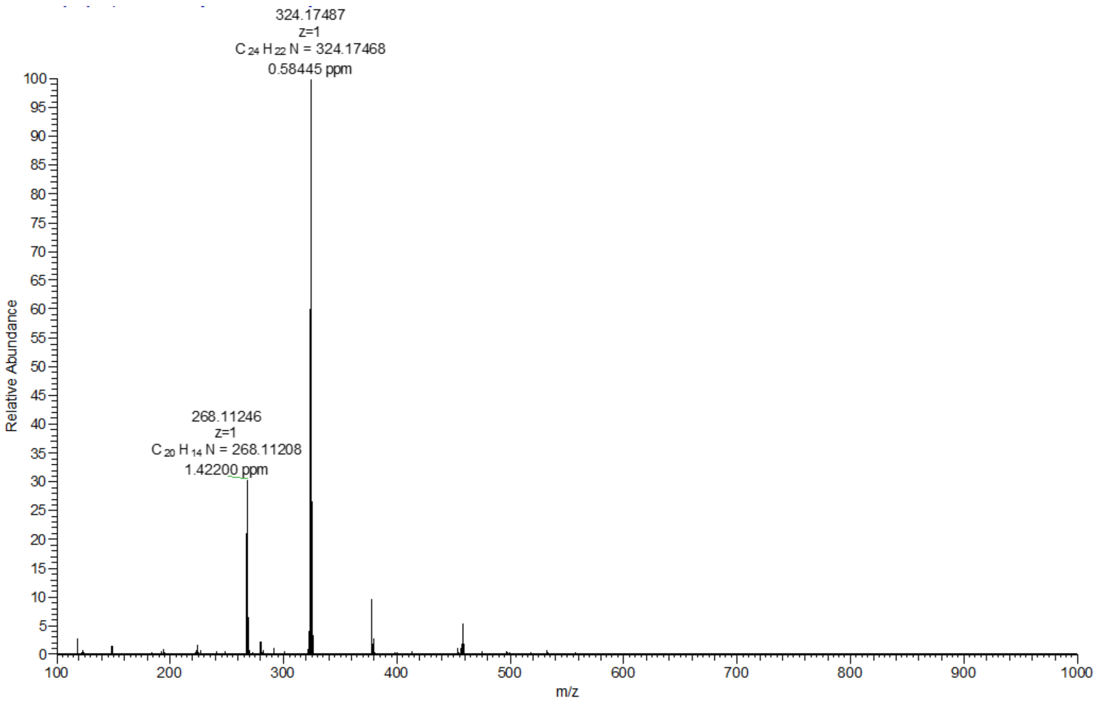

Supplementary Fig. 53. | HRMS spectra. High-resolution mass spectrometry of BCZ-*n*Bu.

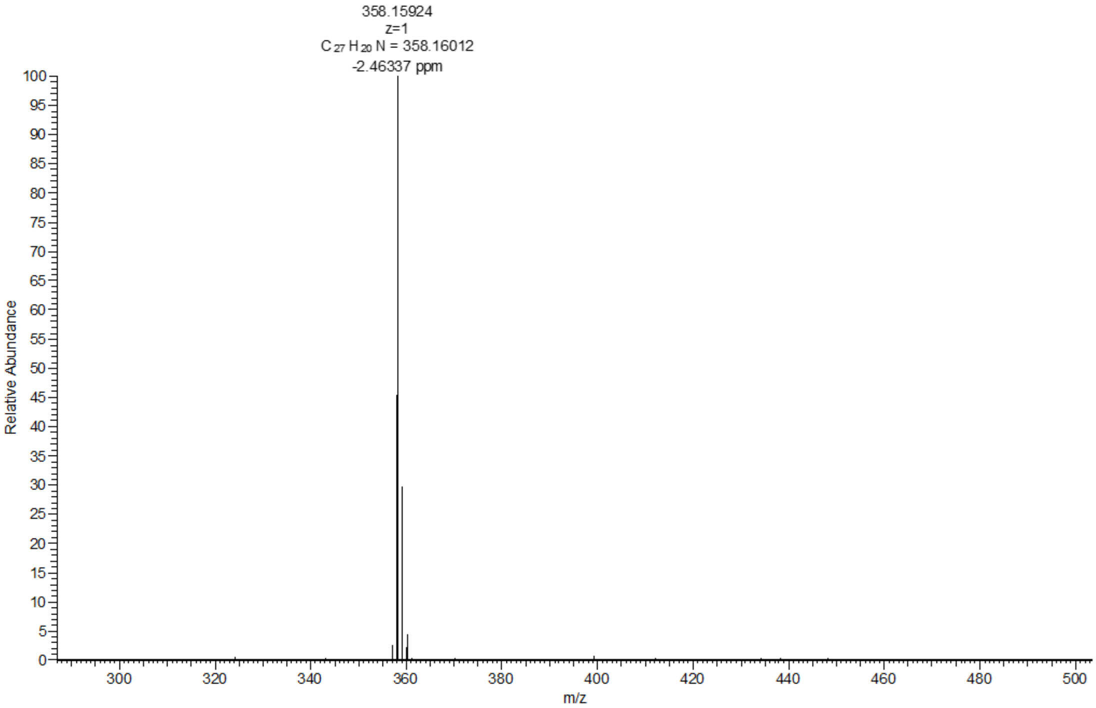

Supplementary Fig. 54. | HRMS spectra. High-resolution mass spectrometry of BCZ-Be.

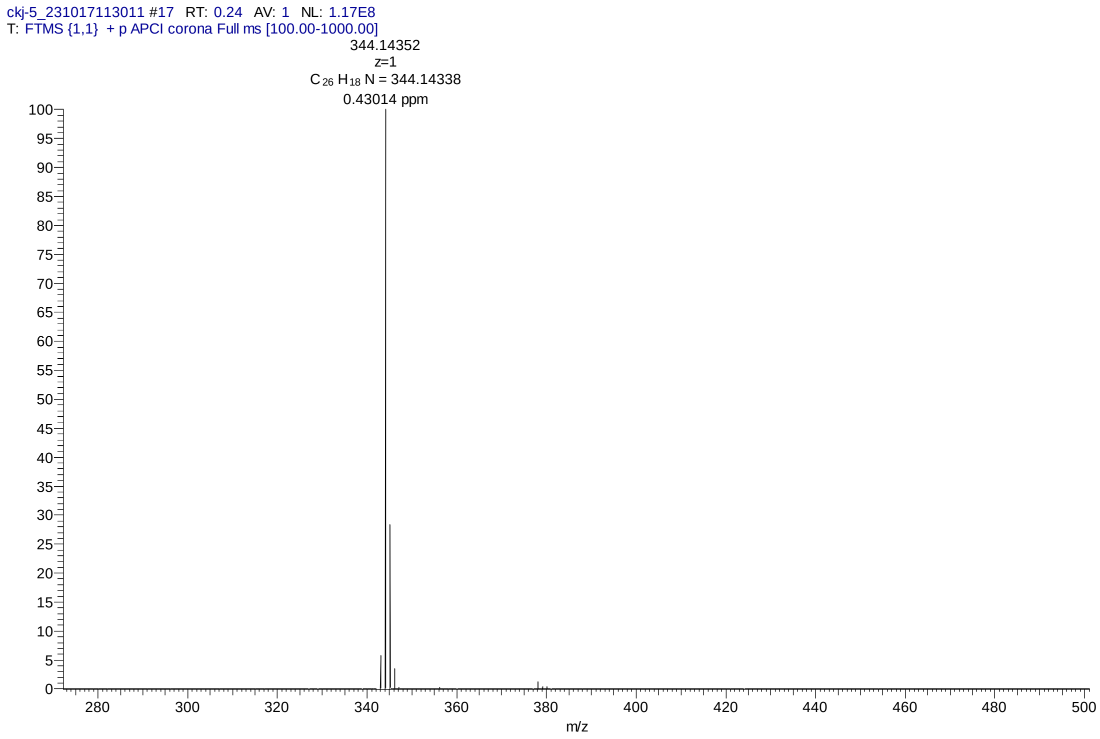

Supplementary Fig. 55. | HRMS spectra. High-resolution mass spectrometry of BCZ-Ph.

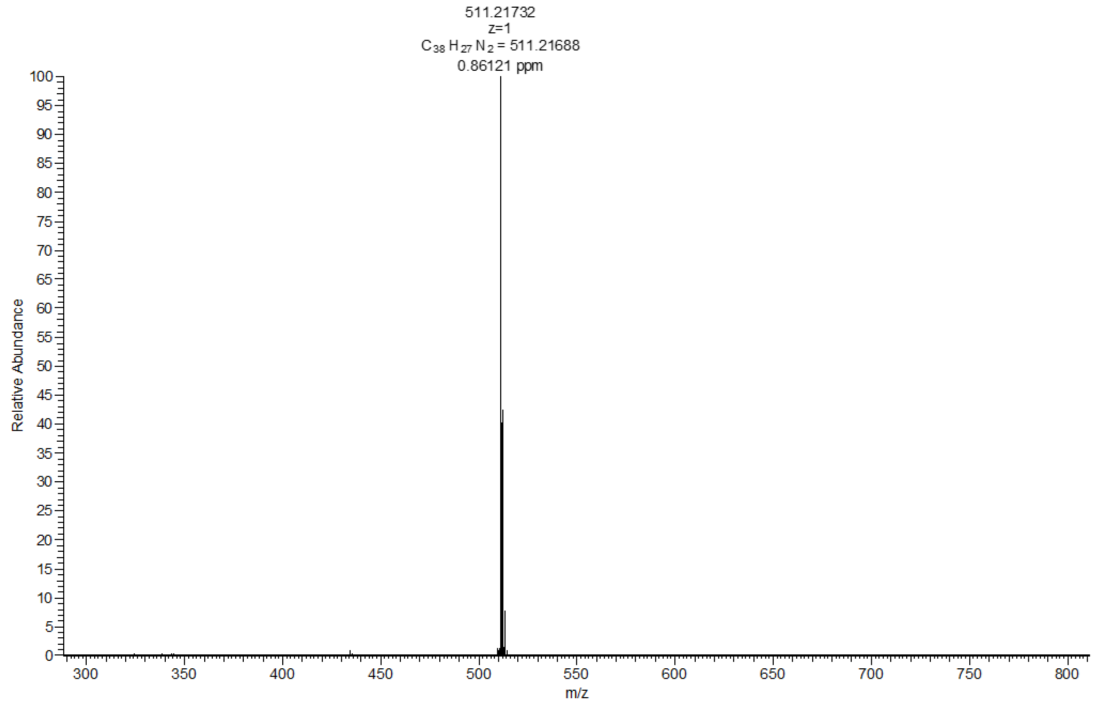

Supplementary Fig. 56. | HRMS spectra. High-resolution mass spectrometry of BCZ-TPA.

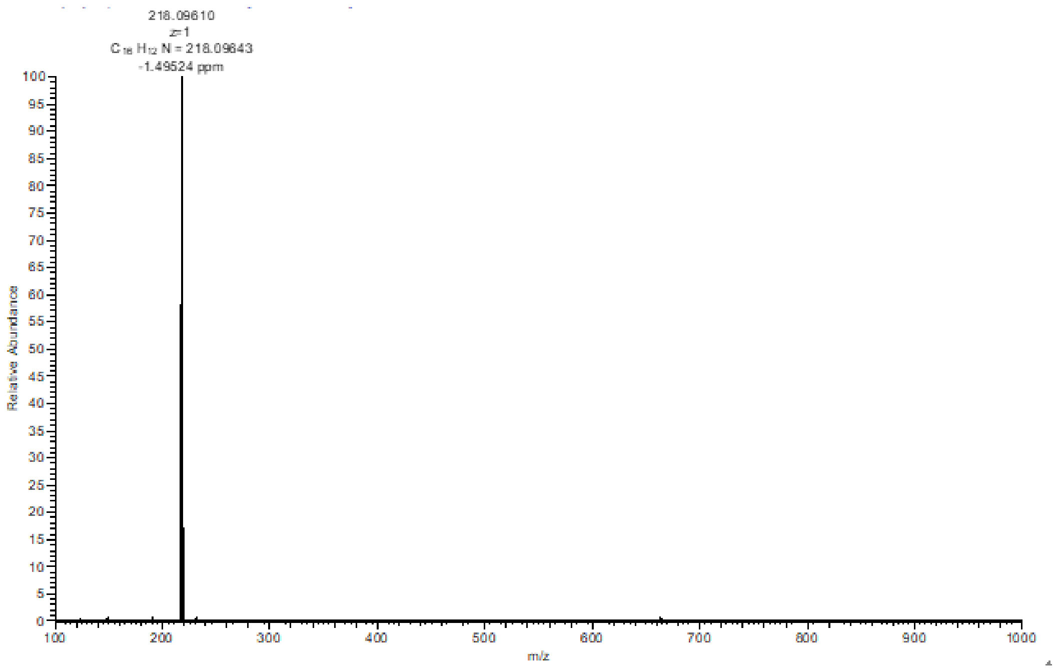

Supplementary Fig. 57. | HRMS spectra. High-resolution mass spectrometry of cBCZ.

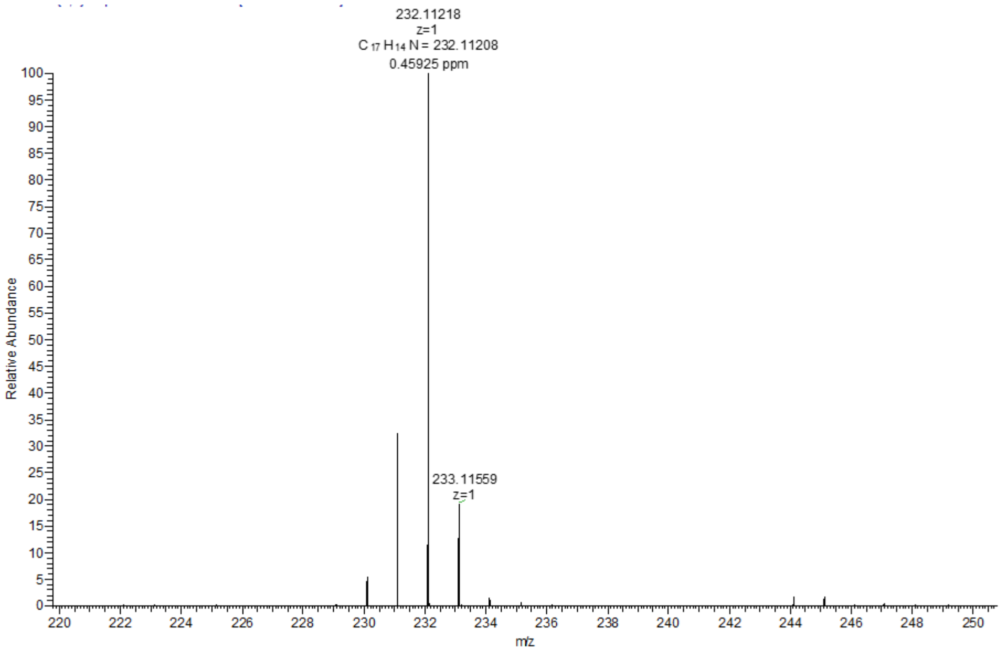

Supplementary Fig. 58. | HRMS spectra. High-resolution mass spectrometry of cBCZ-Me.

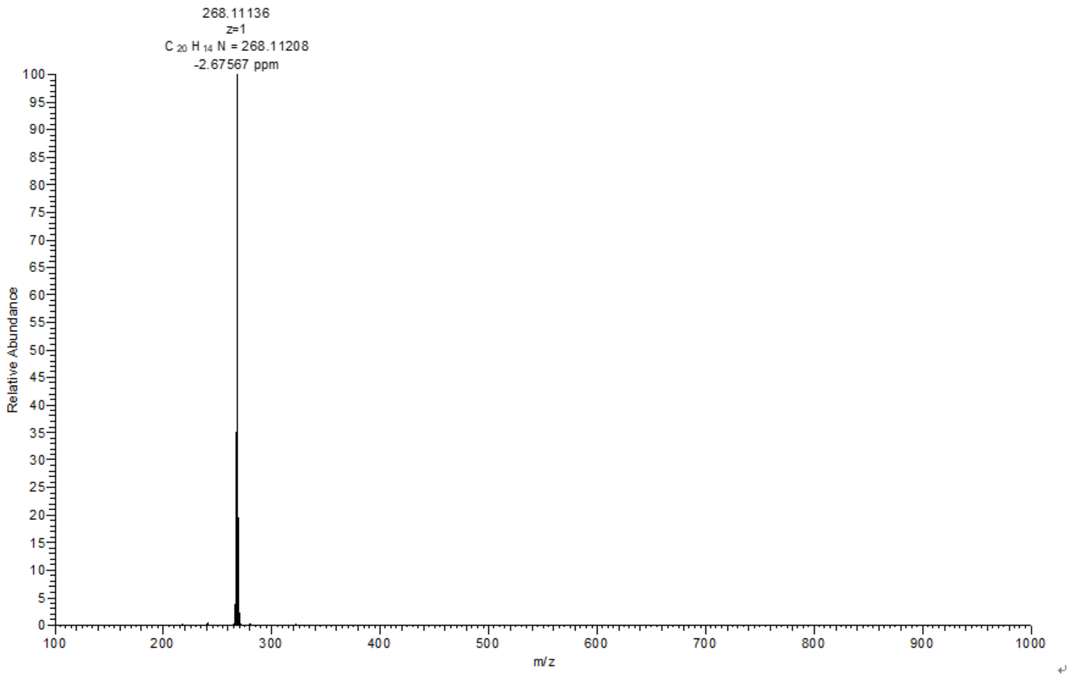

Supplementary Fig. 59. | HRMS spectra. High-resolution mass spectrometry of cgBCZ.

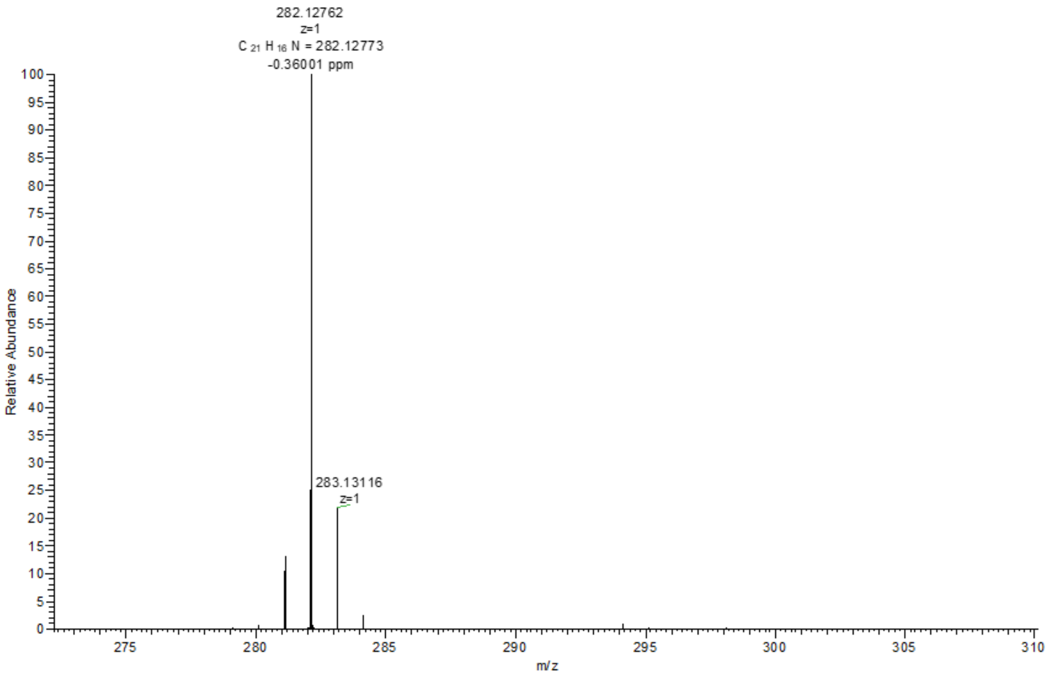

Supplementary Fig. 60. | HRMS spectra. High-resolution mass spectrometry of cgBCZ-Me.

Supplementary References

1. Ye, Z. et al. Cascade π-extended decarboxylative annulation involving cyclic diaryliodonium salts: site-selective synthesis of phenanthridines and benzocarbazoles via a traceless directing group strategy. *Org. Lett.* 21, 9869-9873 (2019).
2. Chen, X. et al. Pd(II)-catalyzed synthesis of polycyclic heteroarenes via an aminopalladation/C-H activation/dealkylation/decarboxylative cyclization cascade. *Org. Lett.* 24, 7282-7287 (2022).
3. Yin, Z. et al. Molecular engineering through control of structural deformation for highly efficient ultralong organic phosphorescence. *Angew. Chem. Int. Ed.* 60, 2058-2063 (2021).
4. Xie, Y. et al. Construction of chiral through-space luminophores via symmetry breaking triggered by sequenced chlorination. *Sci. China Chem*. 66, 2083–2090 (2023).
