# Supplementary material for: Twofold rigidity activates ultralong organic high-temperature phosphorescence
Source: Nat Commun. 2024 Feb 10;15:1269. doi: 10.1038/s41467-024-45678-1 (PMC10858902; doi:10.1038/s41467-024-45678-1)
Supplement: Supplementary file 3 — Description of Additional Supplementary Files [file 41467_2024_45678_MOESM3_ESM.pdf]

Description of Additional Supplementary Files

File Name: Supplementary Data 1
Description: Atomic coordinates of the optimized computational models of six guests.

File Name: Supplementary Data 2
Description: Initial and final configurations of the molecular dynamics trajectories of six guests.
